# Study protocol: Feasibility of medically tailored meals for pediatric populations at risk for disparities in serious illness outcomes due to inequities in food-related social drivers of health (MTM-Kids)

**DOI:** 10.1371/journal.pone.0326762

**Published:** 2025-07-31

**Authors:** Bridgette Thom, Victoria R. Crowder, Andrew B. Smitherman, Bethany S. Cosgrove, Rebecca Bosch, Yashvi Vardhan, C. Natasha Matt, Alice Ammerman, Sheila Judge Santacroce

**Affiliations:** 1 The University of North Carolina at Chapel (UNC) School of Social Work, Chapel Hill, North Carolina, United States of America; 2 UNC Lineberger Comprehensive Cancer Center, Chapel Hill, North Carolina, United States of America,; 3 UNC School of Nursing, Chapel Hill, North Carolina, United States of America; 4 UNC School of Medicine, Chapel Hill, North Carolina, United States of America; 5 UNC College of Arts & Sciences, Chapel Hill, North Carolina, United States of America; 6 UNC Gillings School of Global Public Health, Chapel Hill, North Carolina, United States of America; PLOS: Public Library of Science, UNITED KINGDOM OF GREAT BRITAIN AND NORTHERN IRELAND

## Abstract

**Background:**

Food and nutrition insecurity are actionable, clinically relevant social determinants of health that disproportionately affect pediatric populations, particularly those with serious illnesses, including cancer. Lack of consistent access to nutritious food contributes to poorer treatment tolerance, increased infection risk, lower quality of life, and worsened long-term health outcomes. Medically-tailored meals (MTM) have shown promise in improving health outcomes in adults with diet-sensitive conditions, but their feasibility and acceptability in pediatric populations remain unexplored. The Medically Tailored Meals for Pediatric Populations at Risk for Disparities in Serious Illness Outcomes due to Inequities in Food-Related Social Drivers of Health (MTM-Kids) study aims to assess feasibility of providing medically tailored meals to adolescents undergoing cancer treatment, with a focus on recruitment, retention, parental cost-coping, and preliminary impact on food-related insecurities,.

**Materials and methods:**

This study will enroll 15 adolescent-parent dyads from a pediatric oncology clinic. Participants will receive weekly deliveries of up to 10 frozen medically tailored meals over a 12-week intervention period. Feasibility will be assessed based on recruitment, retention, and adherence to study requirements. Acceptability and appropriateness of the intervention will be evaluated using surveys and semi-structured interviews conducted at 4, 8, and 12 weeks. Secondary outcomes include changes in household food insecurity, financial burden, and parental time demands as well as reported meal satisfaction and chemotherapy-related taste alterations. Quantitative data will be analyzed descriptively, and qualitative data will undergo thematic analysis.

**Discussion:**

The study will provide critical insights into the feasibility of implementing medically tailored meals for pediatric oncology and other patients. Findings will inform the design of a future randomized controlled trial assessing the efficacy of MTM in improving nutritional status, treatment outcomes, and overall well-being in this vulnerable population. MTM-Kids may serve as a scalable intervention to address health disparities related to food insecurity in pediatric populations with serious illness.

**Trial registration:**

ClinicalTrials.gov NCT06814795

## Introduction

*Food insecurity* (uncertainty about having enough food for the household) [1] and *nutrition insecurity* (uncertainty about access and affordability of foods that promote health and well-being) [2] are actionable, clinically relevant social determinants of health (SDOH) for pediatric populations (age 0–17.9 years) [3,4]. Food insecurities are particularly relevant in pediatric populations at risk for disparities in serious illness outcomes [5], including pediatric cancers.

Children living in income poverty with associated adverse SDOH are less likely to survive either highly treatable cancers [6,7] or high-risk cancers that require regimens with costly targeted immunotherapy to optimize prospects for survival [8]. Poor nutrition increases risk of infection and worsens treatment tolerance, quality of life (QOL), daily functioning, and treatment responsiveness [9–12]. Food insecurity may partly account for links between household poverty and poorer outcomes [13,14], and the accelerated biological aging observed in pediatric and young adult cancer survivors [15]. Moreover, poor eating habits developed during treatment can persist into survivorship to heighten risks for secondary cardio-vascular conditions and new cancers [16].

Research on serious illness-related cost coping suggests that parents and other primary caregivers legally and financially responsible for meeting the child’s medical and day-to-day needs (hereafter, “parents”) may stretch food dollars by purchasing low-priced, highly processed food with poor nutritional value, leading to household nutrition insecurity [17,18]. Highly processed foods are often easy to prepare and thus appeal to parents who must juggle the time demands of usual social roles with the complex caregiving often required with serious illness. Highly processed foods may also be appetizing to young patients, who crave foods high in sugar and/or sodium because of chemotherapy-induced alterations in taste [19] or have other common cancer-related symptoms (e.g., fatigue, nausea, pain) that can affect eating [20].

Medically tailored meals (MTM; home-delivered meals tailored to medical needs of individuals with barriers to preparing healthy food) [21] offer a *Food is Medicine* approach to addressing health disparities in pediatric serious illness populations. *Food is Medicine* interventions, which integrate food and healthcare to increase access to nutritious food to support health, include providing: (a) prescriptions for produce (vouchers for food items that may require preparation), (b) medically tailored groceries (food items that may require preparation), and (c) MTMs [22]. In studies of adults with diet-sensitive conditions, MTM interventions were associated with better health outcomes and less health care spending [22]. To our knowledge, however, MTMs have not been tested in pediatric serious illness populations, for which nutrition is a known, important predictor of treatment success, long-term health, and well-being.

The purpose of this study is to assess the potential for scalability of a randomized clinical trial of Medically Tailored Meals for Kids (MTM-Kids), an extension of the MTM concept to pediatric serious illness populations, using pediatric cancer as proof of concept. The MTM-Kids intervention accounts for nutritional needs and chemotherapy-induced alterations in taste among adolescents diagnosed with cancer, and illness-related financial burden and time demands for their parents. The specific aims are to:

1. Determine the feasibility of recruiting a study sample and retaining that sample through the duration of the study period (12 weeks);
2. Determine the feasibility, appropriateness, and acceptability of MTM-Kids from the participants’ perspectives;
3. Explore signals of change in household food-related insecurities, parental cost coping, financial well-being, satisfaction with social role performance;
4. Describe participants’ thoughts about MTM-Kids processes, provided meals, and recommended improvements.

**Fig 1:**
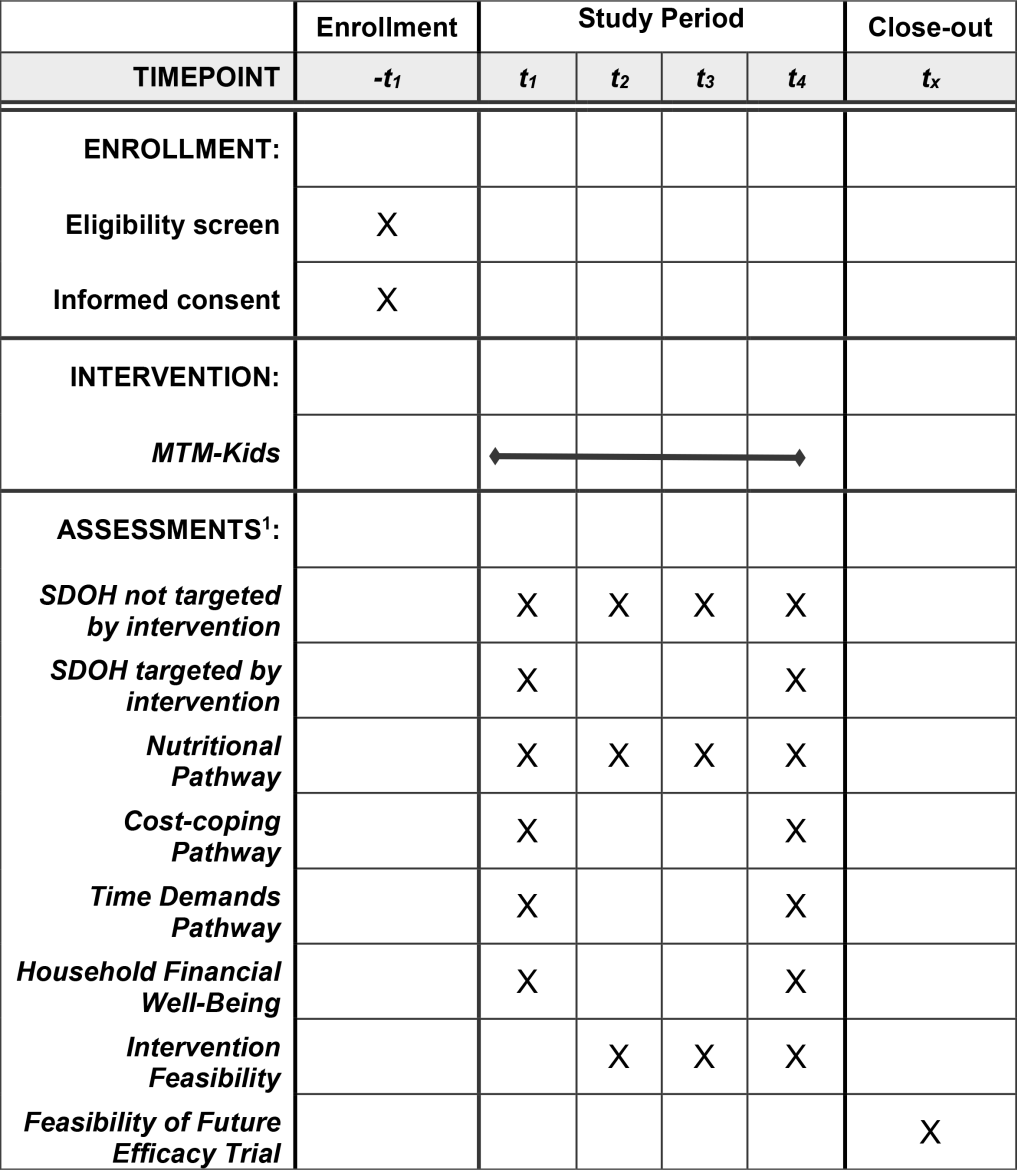
Schedule of enrollment, intervention, and assessments.

We hypothesize that, for households experiencing food-related insecurities, providing healthy frozen meals tailored to common chemotherapy-induced taste alterations is feasible, appropriate, and acceptable to adolescents in active treatment for cancer and their families. (Fig 1).

### Conceptual model

**Fig 2:**
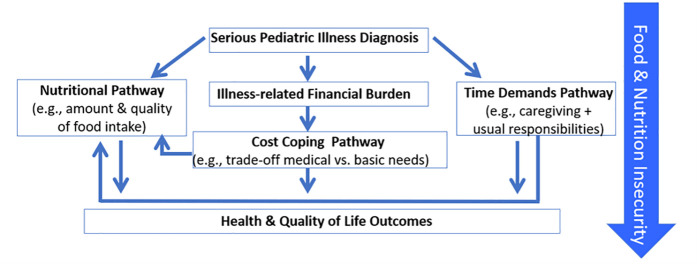
Conceptual model of food and nutrition insecurity in serious pediatric illness (adapted from Santacroce & Kneipp, 2018 and Berkowitz et al., 2023).

Our study is guided by a conceptual model (Fig 2) that posits that children with serious pediatric illnesses who live in households with food-related insecurities are at risk for disparities in health and QOL outcomes by three pathways: (a) illness-related financial burden on household income, which leads to compensatory cost coping (e.g., stretching food dollars by purchasing smaller amounts of foods and low-priced foods which tend to be highly processed); (b) time demands of caregiving during cancer treatment and parents’ other social roles, which leave little time to prepare healthy meals; and (c) physiological manifestations of the serious illness and its treatment on nutrition (e.g., chemotherapy-induced alterations in taste and other eating-related symptoms).

## Materials and methods

### Design and setting

This study, which is guided by the CONSORT extension for feasibility studies [23], applies a longitudinal, multiple methods design to determine the possibility of using the MTM-Kids study processes and intervention in a future full-scale randomized controlled trial. Specifically, we will conduct a single-armed clinical trial of the MTM-Kids intervention, with quantitative and qualitative feedback at multiple time points to enable rapid refinement of the MTM-Kids protocol for a future efficacy trial (Protocol: LCCC2427, 10/06/2024 v1.0). The setting is the pediatric oncology clinic at North Carolina Basnight Cancer Hospital, the only public cancer-specific hospital serving the people of North Carolina.

### Participants

We will focus on children in the adolescent age group (ages 12.0–17.9 years), as they are more likely than younger children to express opinions independent of parental influence [24]. Because sex hormones can affect taste function, processing of sweet and bitter, and food acceptability [25], we will enroll similar numbers of biological females and males to explore the role of biological sex in the quantitative and qualitative data about alterations in taste and meal acceptability.

Adolescents will be eligible for participation if they are willing and able to comply with study procedures based on the judgement of the investigator; are between ages 12.0–17.9 years; use English or Spanish for complex communication; and have completed at least one cycle of cancer chemotherapy and expect to undergo at least 2 more cycles. Primary caregivers of study-eligible adolescents are eligible for participation if they are at least 18 years of age; experience self-reported household material hardship (food, nutrition, utility, or transportation insecurity); and use English or Spanish for complex communication. While we will not ask participants to forgo other forms of food-related assistance to be eligible for the study, we will ask parents to tell us about food assistance requested and received.

### Intervention

For 12 weeks, Equiti Foods, LLC will provide participants with weekly “doses” (meal deliveries) of up to 10 meals per week, as suggested by MTM studies with adult populations [21,22]. A 12-week duration enables three iterations of feedback per participant about his or her experience with MTM-Kids during the prior month (Fig 2). In preliminary efforts, the study team conducted taste testing with adolescents to tailor meal flavorings and textures to taste preferences, preferred meal types and condiments to enable individualization by household members.

### Sample size and sample size calculation

We will enroll five adolescents and one parent per adolescent (five dyads) in the formative test, and then 10 adolescents and 10 parents (10 dyads) in the validation test, for a total of 15 adolescents and 15 parents. The sample size is based on evidence that testing with five users identifies 85% of barriers and facilitators to inform program refinements, and three iterations of feedback per user identifies 98% of barriers and facilitators to accessing and using program materials [26]. Thus, we expect that 15 dyads total, with three iterations of feedback over the 12-week study period, will provide sufficient data to uncover aspects of the study needing refinement prior to future efficacy testing. For the formative and evaluative tests, we will use unique sets of dyads to gain broader perspectives than would be possible with the same sets.

### Study procedures and data collection

**Fig 3:**
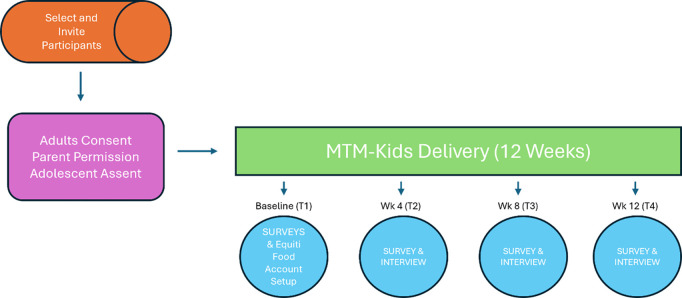
Study schema.

Participation will require (a) completing an online survey at baseline (T1), receipt of MTM-Kids over 12 weeks, (b) participating in interviews at 4, 8, and 12 weeks (T2, T3, T4) after starting to receive meals to inform refinement, and (c) completing a follow-up online survey at 12 weeks (T4). The online survey will be built and the data managed using Research Electronic Data Capture (REDCap), a secure IRB-approved web platform. The study schema is shown in Fig 3.

### Recruitment

A study team member will use electronic health record data and referrals from social workers and other clinic staff to identify eligible dyads (parent endorses at least one household material hardship), connect with the adolescent’s oncology provider, and then monitor the clinic schedule for when the dyad could be approached during a regularly scheduled clinical appointment. Interested dyads will complete the informed consent/assent process and baseline surveys, and then assisted in setting up an account on the Equiti Food, LLC website, where they can select/order the first MTM allotment. Participant-facing study materials will be available in English and Spanish.

After completing T1 and T4 surveys and each interview (T2, 3 and 4), the adolescent and the parent will each receive gift cards ($15 + $15) for a possible total of $150 ($75 for the adolescent + $75 for the parent) per dyad.

Participants who give informed consent, parental permission, or adolescent assent and subsequently withdraw or are removed prior to completing surveys for the primary endpoint may be replaced to obtain the stated number of evaluable participants (N = 15 dyads). The estimated accrual duration is 9 months, and recruitment is anticipated to commence in July, 2025.

### Measures and interviews

Study variables, measures, data sources, and data collection time-points are shown in Table 1. The measures were selected to assess each of the pathways shown in Fig 2 and domains of feasibility. Except for the acceptability, feasibility, affordability and accessibility items, these measures have been used in multiple prior studies of pediatric oncology populations and performed well psychometrically. The measures are specific to adolescents, or to parents.

**Table 1:** Study variables, data source, measures, and data collection time point(s).

Serious illness diagnosis *(health record)*	Cancer type, diagnosis date, treatment protocol	T1
Contact info (parent)	Mobile phone; email address	T1
Demographics (adolescent, parent)	Sex, race, ethnicity, age (birth year)	T1
SDOH not targeted by MTM-Kids (parent)	Education level, marital status, insurance type(s), household income + number of people in household (to estimate % federal poverty level for data collection, primary language used in home	T1
	Address (meal delivery, estimation of neighborhood deprivation), food assistance from other resources	T1-4
SDOH targeted by MTM-Kids (parent)		
• Food insecurity	6-item Food Security Scale^a^	T1, T4
• Nutritional insecurity	4-item Nutrition Security; 4-item Healthfulness Choice^b^	T1, T4
Nutritional Pathway (adolescent)		
• Eating-related symptoms	4-item Basic Taste subscale, Chemotherapy-induced Alterations in Taste Scale^c^	T1-T4
	15-item Self-Reported Symptoms in Pediatrics (SSPedi)^d^	T1-T4
Cost-Coping Pathway (parent)	20-item Financial Coping Behaviors survey^e^	T1, T4
Time-Demands Pathway (parent)	Patient-Reported Outcome Measurement System v.2 (PROMIS v.2) 12-items Satisfaction with perceived social role performance^f^	T1, T4
Household Financial Well-Being (parent)	8-item Personal Financial Well-being Scale^g^	T1, T4
Intervention Feasibility (adolescent, parent)	4-item Acceptability	T2-4
	4-item Appropriateness	T2-4
	4-item Feasibility	T2-4
	Qualitative interviews	T2-4
Feasibility of Future Efficacy Trial	Benchmarks: 70% enrollment, 70% completion of study requirements	Ongoing

SDOH = social determinants of health

^a^United States Department of Agriculture. Household food security survey in the U.S Survey tools – six-item short form of the Food Security survey module. https://www.ers.usda.gov/topics/food-nutrition-assistance/food-security-in-the-us/survey-tools#six

^b^Calloway E, Carpenter L, Gargano T, Sharp J, Yaroch A. Development of new measures to assess household nutrition security and choice in dietary characteristics. *Appetite.* 2022;179:106288.

^c^Kano T, Kanda K. Development and validation of a chemotherapy-induced taste alterations scale. *Oncology Nursing Forum*. 2013; 40(2):e79-85.

^d^Dupuis L, Johnston D, Baggott C, et al. Validation of the symptom screening in pediatrics tool in children receiving cancer treatments. *JNCI: Journal of the National Cancer Institute*. 2018;**110**(6):661−68.

^e^Beauchemin M, Santacroce S, Bona K, et al. Rationale and design of Children’s Oncology Group (COG) Study ACCL20N1CD: Financial distress during treatment for pediatric acute lymphoblastic leukemia in the United States. *BMC Health Services*. 2022;22:832

^f^Hahn E, DeWalt D, Bode RK, et al. PROMIS Cooperative Group. New English and Spanish social health measures will facilitate evaluating health determinants. *Health Psychology. 2014;33(5), 490–499.*

^g^*Prawitz A, Garman E, Sorhaindo B, O’Neill B, Kim J, Drentea P. InCharge Financial Distress/Financial Well-Being Scale: development, administration, and score interpretation. Financial Counseling & Planning*. 2006;17 34–50.

### Adolescents

We will ask adolescents to complete an online survey that includes (a) a brief investigator-developed socio-demographic survey at baseline (T1), (b) established measures of alterations in taste and chemotherapy-induced alterations in taste at weeks 4, 8, and 12 (T2-4) at the start of their semi-structured interviews, and (c) a measure of acceptability and appropriateness at week 12 (T4).

*Adolescent socio-demographic items* include current age (in years), sex at birth, race, their primary language at home, and their current grade (year) in school.

*Chemotherapy-induced alterations in taste* will be measured by 13-items from the Chemotherapy-Induced Alterations in Taste Scale [28]. Adolescents will be asked to use a 5-point scale to indicate how much the taste change bothered them this week. Scores will be calculated by summing item responses and dividing by 13 (lower scores indicate less alteration in taste).

*Other chemotherapy-related symptoms* will be measured by the 15-item Symptom Screening in Pediatrics tool [27]. Adolescents will be asked to use a five-point scale anchored by “0=not at all” and “4=worst bother” to indicate how much each symptom bothered them today or yesterday. Scores will be calculated by summing item responses. Scores can range from 0–60; lower scores indicate less symptom severity.

Semi-structured interviews will focus on the participant’s experience with a study provided meal and their recommendations for improvements.

*Adolescent-reported acceptability and appropriateness* of the MTM-Kids program will be assessed using items that tap into each of these domains. The original items were developed and validated by Weiner and colleagues for used in studies that examine adopters’ perspectives on the acceptability of implementing entire evidence-based programs in practice [29]. For this feasibility study, the items served as the basis for assessing areas that may affect participants’ perspectives on specific aspects of the MTM program. Each domain has four items. Adolescents will be asked to read each item and indicate their response using 5-point scales anchored by “1=completely disagree” and “5=completely agree.” Domain scores will be calculated by summing item responses and dividing the sum by four. Domain score range is 1–4, with higher scores indicating better acceptability or appropriateness.

### Parents

We will ask parents to complete an online survey that includes established measures of household food and nutrition insecurity, time-demands, financial cost-coping behavior and financial wellbeing.

*Household food insecurity* will be measured by the six-item Food Security Scale [30]. Parents will be asked to indicate whether they experienced the condition during the past three months and, in some cases, how often. Scores will be estimated by counting the number of affirmative responses (“yes,” “sometimes,” or “often”); these scores can be categorized, such that 0–1 = very low food insecurity, 2–4 = low food insecurity, and 5–6 = high food insecurity. [30]. Parents will also be asked to indicate if they received assistance with food from government programs (Supplemental Nutrition Program [SNAP], Supplemental SNAP for Women, Infants and Children (WIC), school-provided meals) or community resources (food pantries) within the past six months.

*Household nutrition insecurity* will be measured by the four-item Nutrition Security Scale plus two items from the Healthfulness Choice Scale [31]. Parents will be asked to use a five-point scale, anchored by “1=never” and “5=always,” plus a “don’t know/prefer not to answer” option. Scores will be estimated by counting the number of affirmative responses (“sometimes,” “often,” or “always”); these scores can be categorized such that 0–1 = very low nutrition insecurity, 2–4 = low nutrition insecurity, and 5–6 = high nutrition insecurity [31].

*Parental role time-demands* will be measured with a set of Patient-Reported Outcome Measurement System v.2 (PROMIS v.2) items that ask about satisfaction with perceived social role performance [32]. Parents will be asked to respond to the PROMIS items using a five-point scale anchored by “1=not at all” and “5=very much.” Item responses will be summed and scored using the *t-*score metric. Higher scores indicate greater satisfaction with social role performance [32].

*Financial coping behaviors* will be measured by seven of the questions from the Financial Coping Behaviors Scale currently being asked in a study of financial distress during treatment of pediatric acute lymphoblastic leukemia. [56] Parents participating in MTM-Kids will only be asked about financial coping strategies with implications for the health of the adolescent or another family member. Parents will be asked to use a three-point response scale anchored by “0=never” and “2=several times” to indicate how often they used each coping behavior in the prior month. Item responses will be summed for a total score that can range from 0–14 [33].

*Financial well-being* will be measured by the eight-item Personal Financial Well-being Scale (PFWS) [32]. The PFWS uses a 10-point response scale. Scores are calculated by summing item responses and dividing by eight. Scores can range from 1–10, with “1” indicating “poorest financial wellbeing” and “10” indicating “highest financial wellbeing.” The PFWS has undergone rigorous psychometric testing to establish validity of the latent construct, psychometric properties and norms [34].

*Parent-reported acceptability, appropriateness, feasibility, affordability, and accessibility* of the MTM-Kids program will be measured by items developed by others for use in studies examining the implementation of evidence-based interventions in practice [29] and adapted for this feasibility study. Each domain has four items, and parents will be asked to read each item and indicate their response using five-point scales anchored by “1=completely disagree” and “5=completely agree.” Domain scores will be calculated by summing item responses and dividing the sum by four. The domain score range is 1–4, with higher scores indicating better MTM program acceptability, appropriateness, feasibility, affordability, or accessibility [29].

### Interviews

Adolescents and parents will also be asked to participate in three audio-recorded individual semi-structured interviews via an approved secure platform or in person during the next regularly scheduled clinical visit. Interviews will last 20–30 minutes. Parents will be asked to help their adolescents enter video sessions, although we expect most adolescents will be familiar with this process. A trained team member will conduct the interviews, which will consist of questions that tap into the three pathways that comprise the conceptual model (see Fig 2). First, adolescents will be asked to complete validated measures of cancer-related symptoms [27] and chemotherapy-induced alterations in taste (physiological changes) [28], say which meal they tried (or considered trying), then answer questions about how that worked for them (or did not) and provide recommendations for improvements. Parent interviews will focus on logistics (available selection, ordering, delivery, timing, storage), meal use relative to treatment-related events and associated caregiving demands (time-demands pathway), and adequacy of the allotment (cost-coping pathway). Since they might place meal orders on behalf of their household, we will also ask adolescents to comment on logistics.

### Data management

Home delivery of study provided meals requires that we ask participating parents to provide their name, home address and phone number, which are personal identifiers. To protect against risks to participant privacy, our data management plan, developed in collaboration with an information technology expert at our institution, includes that the study team member will help participants create a profile in the vendor portal using an alias rather than their name, select usernames and passwords, and then share this information with them so participants can place their orders moving forward. We will retain a list of study numbers (not names), aliases, usernames and passwords as a backup. After 12 weeks of food delivery, we will remove the home address from our study records and delete the participant’s profile from the vendor portal.

### Analyses

We will calculate descriptive statistics (frequency, proportion, central tendency, range, standard deviation [SD], reliability) to describe the study sample, estimate proportions, odds ratio, and confidence intervals (CI) for a priori feasibility benchmarks, and assess the psychometric performance of the standardized measures in the study sample.

Feasibility study data can provide unreliable effect size estimates given small sample sizes [35]. Thus, we will follow Brown [36] and estimate SDs and CIs for signals of change in food-related insecurities, cost-coping, time demands and QOL and use upper limits of 80% CIs, plus enrollment and retention data, to estimate sample size for the future efficacy trial. We will also consider evidence for associations between improvement in food security and QOL from studies of MTM with adult populations in estimating sample size for the future trial [11,44].

The primary quantitative endpoint is the feasibility of recruitment and retention of a study sample for a future efficacy trial. Feasibility will be defined as 70% enrollment, 70% completion of baseline and Week 12 surveys, and 70% receipt of at least 4 weeks of meal deliveries and completion of at least one interview by adolescent and by parent (either week 4, 8 or 12). The secondary quantitative endpoint is participant-reported acceptability, appropriateness, feasibility, affordability, and accessibility of the MTM-Kids intervention, defined as mean overall scores for the 4-item measures of each domain ≥ 3 (agree) for 70% of adolescents (acceptability and appropriateness) and 70% of parents (acceptability, appropriateness, feasibility, affordability, and accessibility) at Week 12 (T4).

For qualitative data, within days of an interview, two coders per interview will use rapid qualitative content analysis [37] to analyze verbatim transcripts, compare their results and engage in discussion to resolve any discrepancies. The conceptual model (Fig2) will guide the rapid analysis. The goal is to identify actions at multiple levels (meal, adolescent, household, study) to inform refinements for validation testing and ultimately the protocol for the future trial. The results and any related modifications to the study protocol the study team via secure university-affiliated SharePoint site and submitted to the local protocol review committee and IRB for approval. When all interviews have been completed, we will use traditional content analysis [38] to identify themes in the data overall to inform further refinements.

### Safety and monitoring

The Principal Investigator will provide continuous monitoring of subject safety in this trial with periodic reporting to the comprehensive cancer center’s Data and Safety Monitoring Committee (DSMC). Meetings will be held by teleconference at a frequency dependent on study accrual. These meetings will include the investigators, the project manager, research assistants, and any other relevant personnel the investigators may deem appropriate. At these meetings, the research team will discuss all issues relevant to study progress, including enrollment, safety, regulatory, and data collection.

The project manager will produce summaries or minutes of these meetings. These summaries will be available for review by study team members, and inspection when requested by any of the regulatory bodies charged with the safety of human subjects and data integrity.

The DSMC will review the study on an annual basis at the time of IRB annual review, with the option to exempt from the study from future review. The Principal Investigator (PI) will be responsible for submitting the following information for review: 1) safety and accrual data; 2) significant developments reported in the literature that may affect the safety of subjects or the ethics of the study; 3) preliminary response data; and 4) summaries of team meetings that have occurred since the last report. Findings of the DSMC review will be disseminated by memo to the PI, and institutional Protocol Review Committee, IRB and Data Safety Monitoring Board.

## Discussion

The proposed study is, to our knowledge, the first of its kind to apply a *Food is Medicine* approach to lessening food and nutrition insecurity in a pediatric serious illness population through MTMs specifically tailored to meet the time and financial demands of parents of adolescents with cancer, and address chemotherapy-induced taste changes. MTMs are the most intensive type of *Food is Medicine* intervention, indicated for persons with substantial physical and/or contextual barriers to preparing healthy meals. In studies of adults, MTM interventions were associated with better health outcomes and less healthcare spending [21,22].

To date, in pediatric oncology, the key approach to address outcome disparities driven by food insecurity is to provide the household with gift cards for an online grocery delivery platform [38]. Card value is determined by household size and minimal cost for the Thrifty Food Plan per United States Department of Agriculture [39]; however, no nutritional guidance is provided, and parental time demands may limit their capacity to prepare healthy meals. To address health disparities in households experiencing food insecurities and serious pediatric illness, we need holistic models that embed equity in care delivery by integrating food and nutrition interventions with state-of-the-science clinical care. The proposed study will thus assess the feasibility of such an intervention.

Importantly, our study applies a conceptual model that considers the contextual variables that influence food and nutrition insecurity: illness-related financial burden, time demands on parents, and physiological manifestations of the serious illness and its treatment on nutrition. Our study carefully considers each factor and measures changes to each over time.

### Limitations

Our study will be conducted with recognition of several key limitations. Namely, participants will be drawn from a single institution—a comprehensive cancer center at a public institution—which may limit our ability to generalize our findings. To mitigate this, we draw attention to the broad reach of our public cancer center in serving the entire state of North Carolina as well as our efforts toward language inclusivity. Additional potential limitations are noted in that our cancer center is well-resourced and that the study involves close partnership with an established for-profit organization focused on food and nutrition insecurity, two factors that we acknowledge may not be available broadly.

### Next steps and dissemination plans

This study is a first step towards system-level interventions that embed equity into holistic care delivery for pediatric populations at risk for poorer outcomes of serious illness due to food or nutrition insecurity. Findings will inform a future efficacy trial of the MTM-Kids intervention to mitigate food-related insecurities for households of adolescents in active treatment with chemotherapy for pediatric cancer, with the plan to extend the model to other serious illnesses impacting children and their families.Planned dissemination includes submission of abstracts to national nursing, nutrition, and health services delivery conferences (with topics tailored by discipline). We will also submit two full-length original research reports: the first will be a multiple-methods analysis centerered on the intervention feasibility, acceptability, appropriateness outcomes, and the second will explore outcomes related to our conceptual model.

We will use equitable processes to ensure that all who participated in study co-development and meet criteria for ethical authorship are given proper attribution. These processes will include (a) joint first authorship, (b) promotion of trainee and junior faculty visibility as presenting author at scientific meetings, (c) prioritization of requested grant funds and other available funds for open access publication fees and travel to scientific meetings by graduate students, postdoctoral fellows and junior faculty, and (d) acknowledgement of participants, research staff, community partners and others who contribute to the study through, for example, data collection but do not meet criteria for ethical authorship. Trainees and junior faculty will have access to data for secondary analyses. Each presentation or manuscript will include an attestation about whether, and if so how, artificial intelligence was used in the dissemination process.

## Supporting information

S1 File Santacroce_Protocol_Final. (PDF)

S2 File SPIRIT_Fillable-checklist-15-Aug-2013_Updated 2025-05-07. (PDF)

S3 File SPIRIT-Figure. (DOC)
