# Supplementary material for: Study protocol: Feasibility of medically tailored meals for pediatric populations at risk for disparities in serious illness outcomes due to inequities in food-related social drivers of health (MTM-Kids)
Source: PLoS One. 2025 Jul 31;20(7):e0326762. doi: 10.1371/journal.pone.0326762 (PMC12312939; doi:10.1371/journal.pone.0326762)
Supplement: S1 File — (PDF) [file pone.0326762.s001.pdf]

CLINICAL RESEARCH PROTOCOL

**STUDY NUMBER(S):**	LCCC2427
**PROTOCOL(S) TITLE:**	Feasibility of Medically Tailored Meals for Pediatric Populations at Risk for Disparities in Serious Illness Outcomes due to Inequities in Food-Related Social Drivers of Health (MTM-Kids)
**SPONSOR:**	Lineberger Comprehensive Cancer Center
**ORIGINAL PROTOCOL DATE:**	06 October 2024
**VERSION NUMBER:**	Version 1
**VERSION DATE:**	06 October 2024

LCCC2427
Feasibility of Medically Tailored Meals for Pediatric Populations at Risk for Disparities in Serious Illness Outcomes due to Inequities in Food-Related Social Drivers of Health

Principal Investigator Name (printed): Sheila J. Santacroce

Study Synopsis

Food and nutrition insecurity are clinically relevant, actionable social drivers of health (SDOH) disparities in pediatric populations experiencing serious illnesses, for example, cancer. One approach being studied to address outcome disparities driven by food-related SDOH in the pediatric cancer context is to provide gift cards for an online grocery delivery platform. Prior research on compensatory cost coping with illness-related financial burden suggests that parents may stretch these food dollars by purchasing lower priced food with poor nutritional value. We also know that parents and siblings may cope with illness-related financial burden by going without food and other basics to conserve household assets to meet the needs of the adolescent who is ill, which heightens parent and siblings' risk for health disparities. Medically tailored meals (MTM; home-delivered meals tailored to medical needs of individuals with SDOH + barriers to preparing healthy food) that consider nutrition, illness- related alterations in taste, and parental time demands offer a Food is Medicine approach to addressing food insecurity-related outcome disparities in pediatric serious illness populations. In studies of adults with health conditions, MTM interventions were associated with better health outcomes and less health care spending. We extend the MTM concept to pediatric populations in which inequities in SDOH can affect serious illness outcomes, using pediatric cancer as proof of concept (POC). Our purpose is to determine feasibility of our methods and MTM intervention for a future efficacy trial to improve food and nutrition security for households of children at risk for poorer cancer outcomes due to inequities in food-related SDOH. We will accomplish our objective by collaborating with a small business that makes tasty, healthy frozen meals with start-up funding from National Institute of Minority Health and Health Disparities. Our primary objective is to determine feasibility of recruitment and retention for a future efficacy trial involving an intervention that provides healthy, frozen meals tailored to tastes of children in the adolescent sub-group (ages 12-17.9 years) who are undergoing chemotherapy feasible and acceptable to the adolescents and their primary parental caregiver. We will collect quantitative and qualitative data at multiple time points from adolescents undergoing chemotherapy and their parents to inform rapid, data-driven refinements in our methods and intervention. Our results will inform the potential and protocol for a future full-scale trial and cost effectiveness studies that inform policies extending healthcare financing models to include MTM in pediatric outpatient settings where children with serious illness receive care.

STUDY SUMMARY

**Study Description:**	Food and nutrition insecurity are clinically relevant, actionable social drivers of health (SDOH) disparities in pediatric populations experiencing serious illnesses, for example, cancer. Food and nutrition insecurity may contribute to observed disparities in pediatric cancer outcomes. This study will collect quantitative and qualitative data at multiple time points from children undergoing chemotherapy and their parents to inform rapid, data-driven refinements in our methods and intervention. The results will inform a future full-scale trial and cost effectiveness studies that inform policies extending healthcare financing models to include MTM in pediatric outpatient settings where children with serious illness receive care.
**Type**	Single-arm feasibility study
**Rationale:**	Our primary research question: is an intervention that provides healthy, frozen meals tailored to tastes of children undergoing chemotherapy feasible and acceptable to the children and their primary parental caregiver?
**Target Population:**	Pediatric cancer patients who have received chemotherapy
**Number of Subjects:**	30, including 15 adolescents (ages 12-17.9 years) and 15 parental caregivers (age 18 years or older)
**Estimated Study Duration**	12 months
**Estimated Subject Duration:**	12 weeks

STUDY SCHEMA

Figure 1- Schema

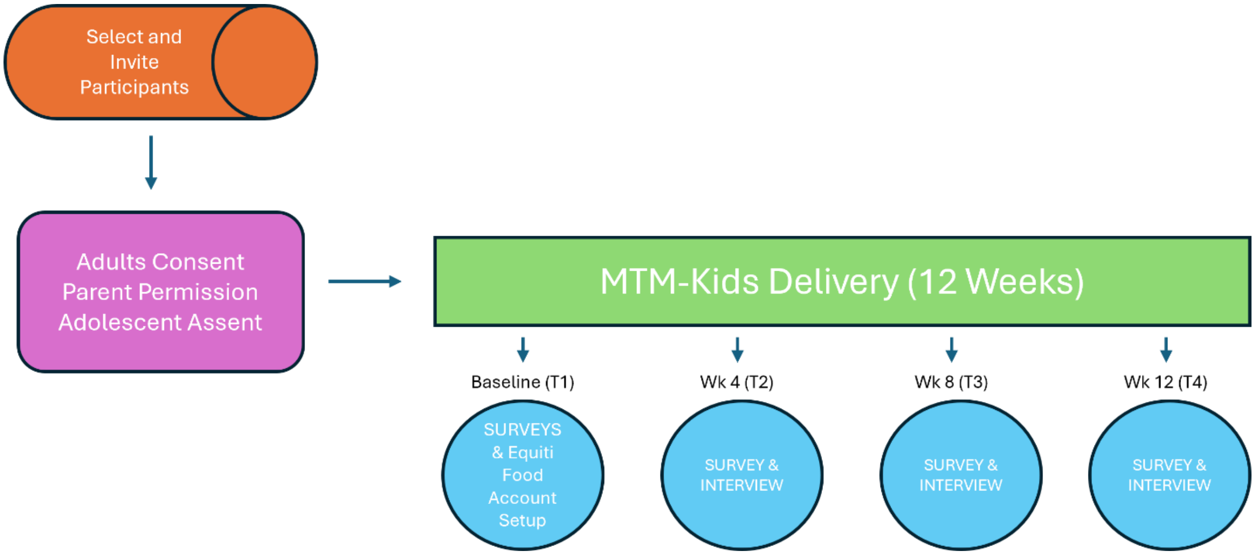

TABLE OF CONTENTS

CONFIDENTIALITY AND INVESTIGATOR STATEMENT 2
STUDY SYNOPSIS 3
STUDY SUMMARY....................................4
STUDY SCHEMA 5
TABLE OF CONTENTS 6
TABLE OF FIGURES....................................9
TABLE OF TABLES....................................9
LIST OF ABBREVIATIONS 10
1 INTRODUCTION AND RATIONALE 11
1.1 Background 11
1.2 Rationale 12
2 STUDY OBJECTIVES 15
2.1 Primary Objective....................................15
2.1.1 To determine feasibility of recruitment and retention for a future efficacy trial of "Medically Tailored Meals-Kids (MTM-Kids)", a food is medicine intervention to mitigate food and nutrition insecurity for households of adolescents in active treatment wth chemotherapy for pediatric cancer .15
2.2 Secondary....................................15
2.2.1 To determine participant-reported acceptability, appropriateness, feasibility, affordability, and accessibility of the MTM-Kids intervention to mitigate food insecurity for households of adolescents in active treatment for pediatric cancer. 15
2.3 Exploratory....................................15
2.3.1 To describe signals of improvement in food insecurity, nutrition insecurity, cost-coping behaviors, parent role satisfaction and financial well-being. 15
2.3.2 To describe participants' thoughts about MTM-Kids processes, the meals, and recommended improvements....................................15
3 STUDY ENDPOINTS 16
3.1 Primary Endpoint 16
3.1.1 Feasibility of recruitment and retention of a study sample for a future efficacy trial of MTM-Kids intervention to mitigate food-related insecurities for households of adolescents in active treatment with chemotherapy for pediatric cancer is defined as 70% enrollment, 70% completion of baseline and Week 12 surveys, and 70% receipt of at least 4 weeks of meal deliveries + completion of at least one interview by adolescent and by parent (either week 4, 8 or 12)....................................16
3.2 Secondary....................................16
3.2.1 Participant-reported acceptability, appropriateness, feasibility, affordability, and accessibility of the MTM-Kids intervention is defined as mean overall scores for the 4 item measures of each domain (appendix X) ≥ 3 (agree) for 70% of adolescents (accetability and appropriateness) and 70% of parents (acceptability, appropriateness, feasibility, affordability, and accessibility) at week 12 (T4). 16

3.3 Exploratory 16
3.3.1 Signals of improvement in food insecurity, nutrition insecurity, cost-coping behaviors, time demands, and financial well-being is defined by improvements in T4 (post intervention) measurements as compared to T1(baseline pre-intervention). 16
3.3.2. 16
4 STUDY PLAN 17
4.1 Study Intervention 17
4.2 Recruitment 18
4.3 Expected Risks 18
4.4 Time & Events Table 19
5 STUDY POPULATION 21
5.1 Inclusion Criteria 21
5.2 Exclusion Criteria 21
5.3 Single Subject Exceptions 21
5.4 Participant Replacement 21
5.5 Inclusion of Women and Minorities 22
6 STUDY CONDUCT 22
6.1 Duration of Intervention 22
6.2 Duration of Follow-up 22
6.3 Participant Withdrawal 22
6.4 Participants Lost to Follow-up 23
6.5 Off-Study Criteria 23
7 DESCRIPTION OF STUDY PROCEDURES 24
7.1 Assessments 24
7.1.1 Study Eligibility Screening 24
7.1.2 Medical History 24
7.1.3 Equiti Foods Registration 24
7.2 Study Surveys and Interviews 24
7.2.1 Surveys and Interview Questions for Adolescents 24
7.2.2 Surveys and Interview Questions for Parents 25
8 STATISTICS 27
8.1 Study Design 27
8.2 Sample Size, Accrual and Duration of Accrual 27
8.2.1 Sample Size and Power 27
8.2.2 Total Accrual 28
8.2.3 Estimated Duration of Accrual 28
8.2.4 Estimated Total Length of Study 28
8.3 Data Analysis Plans 28
8.3.1 Primary Endpoint Analysis 28
For project to be considered successful and feasible in a larger study, we determined the following benchmarks: a) 70% of those approached are enrolled in the study, (b) 10 out of 15 participating parent/adolescent dyads should complete the baseline and week 12 surveys, (c) 10 out of 15 parents receive at least 4 weeks of meal deliveries, and (d) in 10 out of 15 dyads, the adolescent and the parent each complete at least one interview

(week 4, 8, or 12). We will estimate the retention rate and report it with a 95% confidence interval. 28
8.3.2 Secondary Endpoint Analysis 28
8.3.3 Exploratory Endpoint Analysis 28
8.3.4 Interim Analysis 29
9 STUDY MANAGEMENT 30
9.1 Required Documentation 30
9.2 Institutional Review Board (IRB) Approval and Consent 30
9.3 Registration Procedures 30
9.4 Adherence to Protocol 31
9.4.1 Protocol Deviations/Violations 31
9.5 Study Files and Record Retention 32
9.6 Obligations of Investigators 32
9.7 Data and Safety Monitoring Plan 32
10 AUDITING AND MONITORING 34
11 AMENDMENTS 35
12 STUDY DISCONTINUATION 36
13 CONFIDENTIALITY 37
14 REFERENCES 38
15 APPENDICES 42
15.1 APPENDIX I – Names of Study Personnel 42
15.2 APPENDIX II – Study Measures 43
15.2.1 ADOLESCENT SURVEYS 43
15.2.2 PARENTAL SURVEYS 46
15.3 APPENDIX III – Study Semi-Structured Interview Guides 56
15.3.1 Parent Interview Guide 56
15.3.2 Adolescent Interview Guide 58

TABLE OF FIGURES

Figure 1- Schema..5
Figure 2: Conceptual model of food and nutrition insecurity in serious pediatric illness (adapted from Santacroce, & Kneipp, 2018; and Berkowitz, et al., 2023) 12

TABLE OF TABLES

Table 1: Time & Events..19

LIST OF ABBREVIATIONS

AE	Adverse Event
CAPA	Corrective and preventative action plan
CPO	Clinical Protocol Office
CR	Complete Response
CRF	Case Report Form
DSMB	Data Safety Monitoring Board
DSMC	Data Safety Monitoring Committee
FDA	Food and Drug Administration
GCP	Good Clinical Practice
HIPAA	Health Insurance Portability and Accountability Act
IBC	Institutional Biosafety Committee
ICH	International Conference on Harmonization
IRB	Institutional Review Board
LCCC	Lineberger Comprehensive Cancer Center
NCI-CTCAE	National Cancer Institute Common Terminology Criteria for Adverse Events
NIH	National Institutes of Health
OHRE	Office of Human Research Ethics
ORR	Overall Response Rate
OS	Overall Survival
PRC	Protocol Review Committee
SAE	Serious Adverse Event
SAR	Suspected Adverse Reaction
SD	Stable Disease
SOP	Standard Operating Procedure
UNC	University of North Carolina

1 INTRODUCTION AND RATIONALE

1.1 Background

Food insecurity (uncertainty about having enough food for the household)[1] and nutrition insecurity (uncertainty about access and affordability of foods that promotes health and well-being [2] are clinically-relevant, actionable social drivers of health (SDOH) disparities for pediatric (age 0-17.9 years) populations.[3, 4] Food-related insecurities are especially relevant in pediatric populations vulnerable to disparities in outcomes of serious illness (life-threatening illness with taxing symptoms, treatments, and caregiving requirements).[5] Pediatric cancer is an example. Children living in poverty with associated adverse SDOH are less likely to survive either highly treatable cancers,[6, 7] or high-risk cancers that require regimens with costly targeted immunotherapy to optimize prospects for survival.[8] Poor nutrition increases risk of infection[9, 10] and worsens treatment tolerance, QOL, daily functioning and treatment responsiveness.[11], [12], [13] Food-related insecurities may partly account for links between household poverty and poorer outcomes,[14],[15],[16] and for the accelerated biological aging observed in pediatric and young adult cancer survivors.[17, 18] Moreover, poor eating habits developed during treatment can persist into survivorship to heighten risks for secondary cardio-vascular conditions and new cancers.[19]

Research on serious illness-related cost-coping suggests that parents and other primary caregivers legally and financially responsible for meeting the child's medical and day-to-day needs (hereafter "parents") may stretch food dollars by purchasing low priced, processed food with poor nutritional value.[20, 21] These purchasing choices contribute to household nutrition insecurity. Processed foods are easy to prepare and thus appeal to parents who juggle time demands of complex caregiving and usual social roles. Because of chemotherapy-induced alterations in taste, these foods also appeal to young patients who crave foods high in sugar and/or sodium.[22] In pediatric cancer, one approach being studied to address observed outcome disparities driven by food insecurity is to provide the household with gift cards for an online grocery delivery platform. Card value is determined by household size and minimal cost for the Thrifty Food Plan per United States Department of Agriculture (USDA).[23] No nutritional guidance is provided and parental time demands may limit their capacity to prepare healthy meals. To address health disparities in households experiencing food-related insecurities and serious pediatric illness, we need holistic models that embed equity in care delivery by integrating food and nutrition interventions with state-of-the-science clinical care to address adverse SDOH.

Medically tailored meals (MTM) offer a Food is Medicine[24] approach to addressing health disparities in pediatric serious illness populations. MTM comprise home-delivered meals tailored to medical needs of individuals with SDOH-driven health disparities and barriers to preparing healthy food. In studies of adults, MTM interventions were associated with better health outcomes and less health care spending.[24, 25] Here, to our knowledge for the first time, we propose Medically-Tailored Meals for Kids (MTM-Kids), an extension of the MTM concept to pediatric serious illness populations for whom SDOH inequities affect outcomes. We use pediatric cancer as the proof of concept. MTM-Kids account for nutritional needs, chemotherapy-induced alterations in taste, and illness-related financial burden and time demands.

1.2 Rationale

Children with serious pediatric illnesses who live in households with food –related insecurities are at risk for disparities in health and QOL outcomes by three pathways. One pathway of risk is illness-related financial burden, which leads to compensatory cost coping. North Carolina (NC) has shamefully high rates of adolescent poverty; 19.5% of children in NC live in household poverty, and 45% reside in high poverty neighborhoods.[26] Not only do these statistics apply to children newly diagnosed with cancer but an additional 15% of affected households are expected to fall into poverty during the first 6 months following the child's diagnosis.[27] Parental work disruptions are highly prevalent (77%) and, in 30-50% of households, one parent ceases paid work to manage the child's caregiving requirements.[27] Increases in payer-patient cost-sharing (deductibles, co-insurances, co-pays) and out-of-pocket expenditures for healthcare-related travel and lodging near the treatment center contribute to the financial burden.[28] Households with limited English proficiency, low incomes, residences >60 miles/minutes from the treatment center, caregiving-related work disruptions and/or headed by single women are at high risk for catastrophic (>40% of annual household income[29]) illness-related financial burden.[30] This financial burden generates compensatory cost coping whereby parents divert resources ordinarily used to meet basic needs (nutritious food) toward paying medical bills and illness-related out-of-pocket expenses, particularly as funds shrink towards the end of the month and financial burdens do not.[31-33] Among US households reporting food insecurity, 68.4% had incomes in the prior tax year that disqualified them for government food assistance.[34] For qualifying households of patients diagnosed with cancer, their allotted assistance is not adjusted to account for the additional expenses generated by cancer.[35]

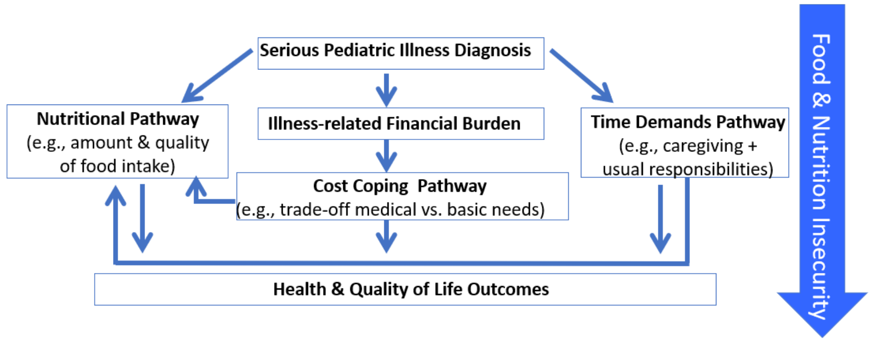

Figure 2: Conceptual model of food and nutrition insecurity in serious pediatric illness (adapted from Santacroce, & Kneipp, 2018; and Berkowitz. et al.. 2023)

A second pathway of risk is time demands on parents. Serious pediatric illnesses are expensive to the healthcare system; the system minimizes its costs by shifting care to outpatient settings and prompting parents to perform clinical tasks (administering medications, flushing catheters, changing dressings, monitoring vital signs, assessing and managing symptoms).[36] Parents must also meet the emotional needs of the adolescent with the diagnosis and any siblings, manage administrative components of their child's care (insurance prior authorization, medical bills, payment plans), maintain their home and communicate with providers and various agencies,[36] all while sustaining some level of employment to support their household and preserve access to employer-sponsored health insurance. These competing demands can similarly contribute to compensatory coping whereby caregivers devote time typically used for usual household tasks, such as grocery shopping and meal prep to caregiving, which affects the nutritional pathway.

Finally, the third pathway of risk is the influence of the physiological manifestations of the serious illness and its treatment on nutrition. Chemotherapy-induced alterations in taste (metallic taste in mouth) and symptoms that affect eating (nausea, vomiting, pain, fatigue, dry mouth) are common and contribute to decline in food-related enjoyment and nutritional intake.[37-41] Taste alterations typically occur within the first week of treatment[40] and last six months or even years following treatment completion.[42] Parents may purchase whatever foods children in active treatment believe they will enjoy (but then the foods do not taste as expected so the adolescent will not eat them), which means less money to buy food for the household. Lengthy outpatient stays require parents to stock both their temporary lodging near the hospital and the family residence with food, which means less capacity for preparing healthy meals and cutting back on food for the household.[43] **To address inequities in household food and nutrition security and thus reduce disparities in outcomes for pediatric serious illness populations, we need rigorous evaluations of high quality, high intensity interventions that comprehensively address these three pathways.**

Food is Medicine interventions (conjunctions of food and healthcare that aim to increase access to nutritious food and thus support health)[44] include offering: (a) prescriptions for produce (vouchers for food items that may require preparation), (b) medically tailored groceries (food items that may require preparation), and (c) MTMs (home-delivered, ready to heat-and-eat meals designed by a registered dietician).[44] MTMs are the most intensive type of Food is Medicine intervention, indicated for persons with substantial physical and/or contextual barriers to preparing healthy meals. Contextual barriers apply to households in which parents navigate the caregiving demands of serious pediatric illness. Short term MTMs (high risk pregnancy), or long-term MTMs (AIDS, diabetes, organ failure) improve outcomes and reduce health care costs for adult populations with SDOH inequities. [25, 44] To our knowledge, MTM interventions have not been tested in pediatric serious illness populations for which nutrition is a known, important predictor of treatment success, long-term health, and well-being. Historically, needs of pediatric populations have received little attention – children lack political clout and thus disparities for pediatric populations persist.[45] This study is a first step towards system-level interventions that embed equity into holistic care delivery for pediatric populations with serious illness. Areas of uncertainty to be addressed by this feasibility study prior to deciding whether to procede with a future definititive trial [46] include the (a) feasibility of recruiting and retaining a sample; (b) feasibility of MTM ordering and delivery, and acceptability, affordability, accessibility, and appropriateness of MTM for the target population, and (c) sensitivity of proposed pathways to MTM.

The goal of this study is to conduct a single-arm study to (a) determine the feasibility of recruiting a study sample and retaining that sample through the duration of the study period (b) determine the feasibility, appropriateness and acceptability of MTM-Kids from the participants' perspectives, and (c) explore signals of change in food-related insecurities, cost coping, time demands and financial well-being. We hypothesize that for households experiencing food-related insecurities, providing healthy, frozen meals tailored to a common chemotherapy-induced taste alteration feasible, is appropriate and acceptable to children in active treatment for cancer and their parents. We will be guided by the CONSORT extension for feasibility studies.[46] Quantitative and qualitative feedback at multiple time points will enable rapid refinements of MTM-Kids and the protocol for the future efficacy trial. Our results will inform the potential for a future 2-group randomized efficacy trial with gift cards for online food delivery as the likely comparator, and later

cost effectiveness studies that advance our goal to extend healthcare financing models to include MTM in pediatric healthcare settings where seriously ill children receive care.

2 STUDY OBJECTIVES

2.1 Primary Objective

2.1.1 To determine feasibility of recruitment and retention for a future efficacy trial of "Medically Tailored Meals-Kids *(MTM-Kids)", a food is medicine intervention to mitigate food and nutrition insecurity for households of adolescents in active treatment with chemotherapy for pediatric cancer*

2.2 Secondary

2.2.1 To determine participant-reported acceptability, appropriateness, feasibility, affordability, and accessibility of the MTM-Kids intervention to mitigate food insecurity for households of adolescents in active treatment for pediatric cancer.

2.3 Exploratory

2.3.1 To describe signals of improvement in food insecurity, nutrition insecurity, cost-coping behaviors, parent role satisfaction and financial well-being.

2.3.2 To describe participants' thoughts about MTM-Kids *processes, the meals, and recommended improvements.*

3 STUDY ENDPOINTS

3.1 Primary Endpoint

3.1.1 Feasibility of recruitment and retention of a study sample for a future efficacy trial of MTM-Kids intervention to mitigate food-related insecurities for households of adolescents in active treatment with chemotherapy for pediatric cancer is defined as 70% enrollment, 70% completion of baseline and Week 12 surveys, and 70% receipt of at least 4 weeks of meal deliveries + completion of at least one interview by adolescent and by parent (either week 4, 8 or 12)

3.2 Secondary

3.2.1 Participant-reported acceptability, appropriateness, feasibility, affordability, and accessibility of the MTM-Kids intervention is defined as mean overall scores for the 4 item measures of each domain (appendix X) ≥ 3 (agree) for 70% of adolescents (acceptability and appropriateness) and 70% of parents (acceptability, appropriateness, feasibility, affordability, and accessibility) at week 12 (T4).

3.3 Exploratory

3.3.1 Signals of improvement in food insecurity, nutrition insecurity, cost-coping behaviors, time demands, and financial well-being is defined by improvements in T4 (post intervention) measurements as compared to T1(baseline pre-intervention).

3.3.2. *Adolescent and parent thoughts about MTM-Kids processes, the meals, and recommended improvements in the meals and study processes are defined as themes that emerge from analyses of qualitative interviews with the adolescents, and with their parents.*

4 STUDY PLAN

This feasibility study uses a longitudinal design and multiple methods to determine the possibility of using the MTM-Kids study processes and intervention in a future full-scale RCT. The study site will be UNC Healthcare's NC Basnight Cancer Hospital Pediatric Oncology Clinic.

4.1 Study Intervention

We will enroll 5 adolescent + 5 parents (5 dyads) in the formative test, and then 10 adolescents + 10 parents (10 dyads) in the validation test for a total of 15 adolescents and 15 parents. The sample size is based on evidence that testing with 5 users identifies 85% of barriers and facilitators to accessing program materials and putting them to use to inform refinements, and 3 iterations of feedback per user during the testing period identifies 98% [47, 48] Thus, we expect that15 dyads total with 3 iterations of feedback via semi-structured interviews with the adolescent and also with the parent over the 12-week study period will rovide sufficient data to uncover aspects of study processes and the program materials that need refinement prior to efficacy testing. For the formative and evaluative tests, we will use unique sets of dyads to gain a broader perspective than is possible with the same sets. We will also focus on children in the adolescent age group (ages 12-17.9 years) for this more in-depth data collection because they are more likely than younger children to express opinions independent of parental influence.[49]

Equiti Foods will tailor flavorings and deliver selected meals along with condiments to enable individualization by household members. The intervention will be 12-weeks duration, with weekly "doses" (meal deliveries) of up to 10 meals per week, as suggested by MTM studies with adult populations. [32, 44] Twelve weeks duration enables three iterations of feedback about the prior month's experience with MTM-Kids.

Participation will require (a) completing a survey at baseline (T1), receipt of MTM-Kids over 12 weeks, (b) participating in interviews at 4, 8, and 12 weeks (T2, T3, T4) after starting to receive meals to inform refinement, and (c) completing a follow-up survey at 12 weeks (T4). While we will not ask participants to forgo other forms of food-related assistance, we will ask them to tell us about food assistance requested and received. Interested dyads will join the informed consent/assent process, complete baseline surveys, and set up an account on Equiti Food's website where they can select/order the first MTM allotment.

Adolescents and parents will also be asked to participate in three audio-recorded individual semi-structured interviews via an IRB-approved secure platform or in person during the next regularly scheduled clinical visit based on their preference. Interviews will last 20-30 minutes. Parents will help adolescents enter video sessions (we expect most adolescents will be familiar with this process from online schooling during the pandemic). A team member with training in clinical care and research of adolescents and families will conduct the interviews. First, adolescents will be asked to complete validated measures of symptoms [50] and chemotherapy induced alterations in taste [51], say which meal they tried (or considered trying), then answer questions about how that worked for them (or didn't) and recommendations for improvements (nutritional pathway). Parent interviews will focus on logistics (meal selection, ordering, delivery, timing), meal use relative to treatment-related events and associated caregiving demands (time-demands pathway), and allotment adequacy (cost-coping pathway).

4.2 Recruitment

Upon IRB approval of the study and requested waiver to access clinical records, we will monitor the clinic schedule for visits by eligible children. A research team member will use EPIC data and referrals from the clinical social workers to identify dyads to contact, connect with the child's oncology provider, and send information about the study to parents via their child's EMR patient portal plus approach the dyad during the clinical appointment. Provided information will be in English and Spanish and include a summary of the study purpose. After completing T1 and T4 surveys and each interview (T2, 3 and 4), the adolescent and the parent will each receive gift cards ($15 + $15) for a possible total of $150 ($75 for the adolescent + $75 for the parent) per dyad.

4.3 Expected Risks

The primary risk for this study is psychological stress and breach of confidentiality, which can cause emotional distress or embarrassment. The study team will have processes in place to protect the security of all study data. All staff will have completed the training pertaining to protecting subject data, and all data will be stored on secured servers in password protected files.

4.4 Time & Events Table

Table 1: Time & Events

Study Assessments	Pre-Study/ Screening[1]	Baseline (T1)[2]	MTM-Kids Meal Deliveries[3]		
			4 weeks (T2)	8 weeks (T3)	12 weeks [illegible]
Eligibility Verification[1]	×				
Informed Adult Consent/Parent Permission/Adolescent Assent/HIPAA Authorization[2]	×				
Equiti Foods Registration[3]		×			
Adolescent Survey[4]					
- Socio-demographics (5 items)		×			
- Symptoms (14 items)			×	×	×
- Chemotherapy-induced alterations in taste (13 items)			×	×	×
- Acceptability (2 items) and appropriateness (2 items)					×
Parent Survey[5]					
- Socio-demographics (15 items)	×				×
- Food (6 items) and nutrition (6 items) insecurity	×				×
- Financial coping behaviors (18 items)	×				×
- Personal Financial Wellbeing Scale (8 items)	×				×
- Participation in social roles and activities (12 items)	×				×
- Acceptable (4 items), appropriate (4 items), feasible (4 items), affordable (4 items), accessible (4 items)	×				×
Medical record abstraction[6]		×			
Gift Card[7]		×	×	×	×
Meal Order and Delivery Process Check[8]		×			
Interview[9]			×	×	×

Footnotes to Time & Events Table

1. Screening includes tests to confirm eligibility.

2. Informed consent will include (a) adult consent from the parent for their participation in the study, (b) parental permission for their adolescent's participation, and (c) adolescent assent. HIPAA authorization to extract a

limited set of data from the adolescent’s electronic medical record. Completion of the parent contact form, baseline assessments and Equiti Food account set-up will commence after the written consent/permission/assent have been obtained.

3. Parent participants must register with the Equiti Foods Website in order to select and order MTM allotment. Allotment will be weekly (up to 10 meals per week), for 12 weeks. Delivery will take 3-5 days and parents can order up tp 5 working days in advance.

4. Adolescents will complete surveys at baseline, T2, T3 and T4. See ADOLESCENT SURVEYS

5. Parents will complete surveys at baseline and at 12 weeks (T4). See PARENTAL SURVEYS

6. A limited set of data (specified in the HIPAA authorization form) about the adolescent’s cancer diagnosis, diagnosis date, treatment protocol, treatment initiation date, and treatment exposures to date will be extracted from the adolescent’s electronic medical record.

7. Gift cards will be given following completion of the Baseline and then the 12-week survey, and following completion of the Week 4, 8 and 12 interviews. The gift cards value will be $15 for parent and $15 for the child, for a possible total of $75 for parent and $75 for child.

8. Parents will be contacted within 1 week following baseline data collection to verify that they have placed the initial meal order, and problem-solve as indicated.

9. Semi-structured interviews will be conducted and audio-recorded via an IRB-approved secure video platform, or in-person during a regularly scheduled clinical visit according to the participant’s preference. An investigator-developed interview guide will inform the interviews (see APPENDIX III – Study Semi-Structured Interview Guides).

5 STUDY POPULATION

To participate in this study, participants must meet ALL eligibility criteria outlined below.

5.1 Inclusion Criteria

1. Written informed parental permission plus adolescent assent to participate in the study plus HIPAA authorization for release of personal health information.

2. Adolescent is willing and able to comply with study procedures based on the judgement of the investigator.

3. Adolescent is age 12-17 years and can use English for complex communication

4. Adolescent has completed at least one cycle of cancer chemotherapy and expects to undergo at least 2 more cycles.

5. Eligible parents of adolescent subjects:

    - Aged 18 years or older

    - Primary caregiver of a study-eligible adolescent

    - Screens positive for household material hardship (food, nutrition, utility, or transportation insecurity)

    - Uses English or Spanish for complex communications

5.2 Exclusion Criteria

1. Adolescent or parent uses language other than English or Spanish to communicate

5.3 Single Subject Exceptions

Eligibility single subject exceptions are not permitted for Lineberger Comprehensive Cancer Center Investigator Initiated Trials under any circumstances. Once a subject is eligible and enrolled, other types of single subject exceptions may be allowed if proper regulatory review has been completed in accordance with Lineberger Comprehensive Cancer Center's Single Subject Exceptions Policy.

5.4 Participant Replacement

Participants who provide informed consent, parental permission, or adolescent assent and subsequently withdraw or are removed prior to meeting the requirements of evaluability for the primary endpoint may be replaced to obtain the stated number of evaluable participants for the study.

5.5 Inclusion of Women and Minorities

The total number of subjects involved in the study will be 30, including 15 adolescents and 15 parents. Of those, we estimate that approximately 50% will be females. The estimated racial/ethnic breakdown of the study may approximately be that 15% may be Black or African American, 4% may be Asian or Native Hawaiian-Pacific Islander. Approximately 18% of the subjects may be Hispanic. While this provides only an estimated breakdown of the study population, efforts will be made to ensure equitable recruitment of individuals that meet the above eligibility requirements. Participants who identify as African American or Hispanic may be over-represented in the study sample compared to the clinical population - children who are African American or Hispanic are 2-3 times more likely to live in households with food insecurity than non-Hispanic White children [26].

6 STUDY CONDUCT

6.1 Duration of Intervention

Intervention may continue for 12 weeks or until:

- Either the adolescent or parent decides to withdraw from study treatment.
- General or specific changes in the child's condition render them unacceptable for further participation in the judgment of the investigator.
- Significant study intervention or activity non-compliance.

If the subject is removed from the intervention, the Principal Investigator should be notified. The reason for discontinuation of a protocol intervention will be documented in the medical records and on the eCRF. In the case where an adolescent or parent decides to prematurely discontinue protocol intervention ("refuses intervention"), the adolescent or parent should be asked if they may still be contacted for further scheduled study assessments. The outcome of that discussion should be documented in the medical records and in the eCRF.

6.2 Duration of Follow-up

Subjects will not be followed after completion of the final survey.

6.3 Participant Withdrawal

If an adolescent or parent decides to withdraw from the study (and not just from the protocol intervention) an effort should be made to complete and report study assessments as thoroughly as possible. At the time of withdrawal, the investigator should attempt to establish as completely as possible the reason for the study withdrawal.

- A complete final evaluation at the time of the participant's study withdrawal should be obtained with an explanation of why the participant is withdrawing from the study.

- If the participant is noncompliant and does not show up for an end of study follow up assessment, this should be documented in the eCRF.

Excessive participant withdrawals from protocol intervention or from the study can render the study not feasible therefore, unnecessary withdrawal of participants should be avoided.

6.4 Participants Lost to Follow-up

A participant will be considered lost to follow-up if they fail to return for three scheduled study visits and is unable to be contacted by the study site staff.

The following actions must be taken if an adolescent or parent participant doesn't show up for a required study data collection:

- The site will attempt to contact the parent and reschedule the missed visit and counsel the subject on the importance of maintaining the assigned visit schedule and ascertain if the participant wishes to and/or should continue in the study.

- Before a participant is deemed lost to follow-up, the investigator or designee will make every effort to regain contact (where possible, 3 telephone calls and, if necessary, a certified letter to the participant's last known mailing address or local equivalent methods). These contact attempts should be documented in the child's medical record or study file.

- Should the parent continue to be unreachable, they will be considered to have withdrawn from the study with a primary reason of lost to follow-up.

6.5 Off-Study Criteria

Participants will be considered off study and no longer be followed on the protocol for any reason if any of the following criteria are met:

- When the child-parent dyad reaches the end of the study as defined by the Time & Events Table

- Either member of the dyad withdraws consent for treatment and any further data collection

- Death

- Lost to follow-up (refer to section 6.4)

7 DESCRIPTION OF STUDY PROCEDURES

7.1 Assessments

Study assessments will be performed at the times outlined in the Time & Events Table.

7.1.1 Study Eligibility Screening

Referred parents will be screened for study eligibility. Screening for household material hardships will use the approach described by Umaretiya and colleagues [52] in the development of their National Cancer Institute (NCI)-funded health equity intervention (R01CA267107; MPIs: K. Bona & A. Rosenberg).

7.1.2 Medical History

When consent/permission, and assent have been obtained and the HIPAA document signed, a focused medical history concerning the treatment protocol and treatment exposures to date will be performed through extraction of a limited set of data from the adolescents' electronic medical record.***Equiti Foods Registration***

At enrollments, parents will be assisted with establishing an account at enrollment, and then helping the parent sign-in to confirm that the account exists.

7.2 Study Surveys and Interviews

Blank copies of the study measures are in APPENDIX II – Study Measures, and the semi-structured interview guides are in APPENDIX III – Study Semi-Structured Interview Guides. The study survey will include socio-economic items and established measures. Study variables, sources, measures, data collection time-points are shown in Time & Events Table. The measures were selected to assess each of the pathways shown in Figure 1 and domains of feasibility. Except for the acceptability, feasibility, affordability and accessiblility items, the study measures have been used in multiple prior studies of pediatric oncology populations and performed well. [50, 51, 56]

7.2.1 *Surveys and Interview Questions for Adolescents*

Adolescents will be asked to complete (a) a brief investigator-developed socio-demographic survey at baseline (T1), (b) established measures of alterations in taste and chemotherapy induced alterations in taste at weeks 4, 8, and 12 (T2-4) at the start of their semi-structured interviews, and (c) a person-reported measure of acceptability and appropriateness at week 12 (T4).

Socio-demographic items will ask the adolescent to tell us their current age (in years), sex at birth, race, the language they mainly use at home, and their current grade (year) in school.

Chemotherapy-induced alterations in taste will be measured by 13-items from the Chemotherapy-Induced Alterations in Taste Scale (CiTAS) [50]. Adolescents will be asked to use a 5-point scale to indicate how much the taste change bothered them this week. Scores will be calculated by summing item responses and dividing by 13 (lower scores indicate less alteration in taste).

Other chemotherapy-related symptoms will be measured by the 15-item Symptom Screening in Pediatrics (SSPedi) tool. [51] Adolescents will be asked to use a 5-point scale anchored by 0 (not at all) and 4 (worst bother) to indicate how much each symptom bothered them today or yesterday. Scores will be calculated by summing item responses. Scores can range from 0-60; lower scores indicate less symptom severity.

Semi-structured interviews will focus on the adolescent's experience with a study provided meal and their recommendations for improvements.

Adolescent-reported acceptability and appropriateness of the MTM-Kids program will be assessed using items that tap into each of these domain. The orginal items were developed and validated by Weiner and colleagues for used in studies that examine adopters' perspectives on the acceptability, etc. of implementing entire evidence-based programs in practice.[52] For this feasibility study, the Weiner, et al. items served as the basis for assessing areas that may affect participants' perspectives on specific aspects of the MTM program. Each domain has 4-items. Adolescents will be asked to read each item and indicate their response using 5-point scales anchored by 1=completely disagree and 5=completely agree. Domain scores will be calculated by summing item responses and dividing the sum by 4. Domain score range is 1-4, with higher scores indicating better acceptability or appropriateness.

7.2.2 Surveys and Interview Questions for Parents

Parents will be asked to complete established measures of household food and nutrition insecurity, time-demands, financial cost-coping behavior and financial wellbeing,

Household food insecurity will be measured by the 6-item Food Security Scale [53]. Parents will be asked to indicate whether they experienced the condition during the past 3 month and, in some cases, how often. Scores will be estimated by counting the number of affirmative responses (yes, sometimes, or often); these scores can be categorized, such that 0-1=very low food insecurity, 2-4= low food insecurity, and 5-6=high food insecurity. [53]

Household nutrition insecurity will be measured by the 4-item Nutrition Security Scale plus 2 items from the Healthfulness Choice Scale. [54] Parents will be asked to use a 5-point scale, anchored by 1=never and 5=always, plus a don't know/prefer not to answer option. Scores will be estimated by counting the number of affirmative responses (sometimes, often, or always); these scores can be categorized such that 0-

1=very low nutrition insecurity, 2-4= low nutrition insecurity, and 5-6=high nutrition insecurity. [54]

Parental role time-demands will be measured with a set of Patient-Reported Outcome Measurement System v.2 (PROMIS v.2) items that ask about satisfaction with perceived social role performance. [55] Parents will be asked to respond to the PROMIS items using a 5-point scale anchored by 1=not at all and 5=very much. Item responses will be summed and scored using the T-score metric. Higher scores indicate greater satisfaction with social role performance. [55]

Financial coping behaviors will be measured by 7 of the questions from the Financial Coping Behaviors Scale currently being asked in a study of financial distress during treatment of pediatric acute lymphoblastic leukemia (ALL; ACCL20N1CD). [56] Parents participating in MTM-Kids will only be asked about financial coping strategies with implications for the health of the adolescent or another family member. Parents will be asked to use a 3-point response scale anchored by 0=never and 2=several times) to indicate how often they used each coping behavior in the prior month. Item responses will be summed for a total score that can range from 0-14.[56]

Financial wellbeing will be measured by the 8-item Personal Financial Well-being Scale (PFWS). [57] The PFWS uses a 10-point response scale. Scores are calculated by summing item responses and dividing by 8. Scores can range from 1 to 10, with 1 indicating poorest financial wellbeing and 10 indicating highest financial wellbeing. The PFWS has undergone rigorous psychometric testing to establish validity of the latent construct, psychometric properties and norms. [57]

Parent-reported acceptability, appropriateness, feasibility, affordability, and accessibility of the MTM-Kids program will be measured using of the MTM-Kids program will be measured using items developed by others for use in studies examining concerned with the implementation of evidence-based interventions in practice [52] and adapted for this feasibility study. Each domain has 4-items. Parents will be asked to read each item and indicate their response using 5-point scales anchored by 1=completely disagree and 5=completely agree. Domain scores will be calculated by summing item responses and dividing the sum by 4. Domain score range is 1-4, with higher scores indicating better MTM program acceptability, appropriateness, feasibility, affordability, or accessibility. [52]

8 STATISTICS

We will calculate descriptive statistics (frequency, proportion, central tendency, range, standard deviation [SD], reliability) to describe the study sample and estimate proportions, odds ratio, and confidence intervals (CI) for a priori feasibility benchmarks (see Time & Events Table). Feasibility study data can provide unreliable effect size estimates given small sample sizes. [46, 58] Thus, we will follow Brown [59] and estimate SDs and CIs for signals of change in food-related insecurities, cost-coping, time demands and QOL and use upper limits of 80% CIs plus enrollment and retention data to estimate sample size for the future efficacy trial. We will also consider evidence for associations between improvement in food security and QOL from studies of MTM with adult populations in estimating sample size for the future trial. [11, 44] Within days of an interview, two coders per interview will use rapid qualitative content analysis[69] to analyze verbatim transcripts, compare their results and engage in discussion to resolve any discrepancies. The conceptual model (Figure 1) will guide the rapid analysis.[60] The goal is to identify actions at multiple levels (meal, child, household, study) to inform refinements for validation testing and ultimately the protocol for the future trial. When all interviews have been completed, we will use traditional content analysis[56] to identify themes in the data overall to inform further refinements.

To ensure a rigorous and reproducible study, we have taken the following steps:

- Established a plan to enroll a diverse sample
- Justified the sample size
- Selected established measures to measure study variables
- Established the data analysis plan prior to data acquisition
- Considered relevant biological variables

Because sex hormones can affect taste function, processing of sweet and bitter, and food acceptability, [61] we will enroll similar numbers of biological female and male adolescents for each aim and explore the role of biological sex in the quantitative and qualitative data about alterations in taste and meal acceptability.

8.1 Study Design

This feasibility study uses a longitudinal design and multiple methods to determine the possibility of using the MTM-Kids study processes and intervention in a future full-scale RCT. 15 parent/adolescent dyads will be enrolled and asked to complete a survey at baseline (T1), receive MTM-Kids meals over 12 weeks, participate in interviews at 4, 8, and 12 weeks (T2, T3, T4) after starting to receive meals to inform refinement, and complete a follow-up survey at 12 weeks (T4).

8.2 Sample Size, Accrual and Duration of Accrual

8.2.1 Sample Size and Power

The target sample size is 30 (15 adolescents + 15 parents). Sample size for this feasibility study is not based on an effect size, but rather is chosen based on logistic and financial considerations as well as the expertise of the researchers. The sample size is based on evidence that testing with five users identifies 85% of barriers and facilitators

to inform refinements, and three iterations of feedback per user during the testing period identifies 98% of barriers and facilitators to inform needed improvements in study processes and the intervention.

8.2.2 Total Accrual

The total accrual will be 30 (15 adolescents + 15 parents).

8.2.3 Estimated Duration of Accrual

The estimated accrual duration is 9 months

8.2.4 Estimated Total Length of Study

The estimated total study length is 12 months.

8.3 Data Analysis Plans

8.3.1 Primary Endpoint Analysis

For project to be considered successful and feasible in a larger study, we determined the following benchmarks: a) 70% of those approached are enrolled in the study, (b) 10 out of 15 participating parent/adolescent dyads should complete the baseline and week 12 surveys, (c) 10 out of 15 parents receive at least 4 weeks of meal deliveries, and (d) in 10 out of 15 dyads, the adolescent and the parent each complete at least one interview (week 4, 8, or 12). We will estimate the retention rate and report it with a 95% confidence interval.

8.3.2 Secondary Endpoint Analysis

Descriptive statistics (mean, standard deviation [SD], score range) of the measures of adolescent acceptability and appropriateness as well as parental acceptability, appropriateness, feasibility, affordability and accessibility at week 12 will be reported.

8.3.3 Exploratory Endpoint Analysis

Descriptive statistics (mean total scores, score ranges, standard deviations) and figures will be used to portray the results of baseline (T1) and 12 weeks (T4) assessments of household food insecurity, household nutrition insecurity, parental financial coping strategies, parental time demands, and parental financial wellbeing.

Descriptive statistics (mean total score, score range, SD) swill be used to portray adolescents’ treatment-related symptoms and chemotherapy-induced alterations in taste.

Qualitative descriptive approaches will be used to identify themes in the interviews with adolescents and parents about their experiences with MTM-Kids meals and recommendations for needed improvements.

Treatment exposures, scores on assessments of symptom and chemotherapy induced alterations in taste will be used to qualitatively explore linkages between symptoms, alterations in taste, and themes in the interview data.

8.3.4 Interim Analysis

When 5 adolescent-parent dyads (formative test) have completed study requirements, their data will be analyzed as described above, study processes and the MTM-Kids intervention will be refined as needed, and then the validation test will commence.

9 STUDY MANAGEMENT

9.1 Required Documentation

Before the study can be initiated at any site, the following documentation must be provided to the University of North Carolina.

- A copy of the official IRB approval letter for the protocol and informed consent
- IRB membership list or Federalwide Assurance (FWA) number.
- CVs and medical licensure for the Principal Investigator and any sub-investigators who will be involved in the study.
- The Investigator's signature documenting understanding of the protocol and providing commitment that this trial will be conducted according to all stipulations of the protocol.

9.2 Institutional Review Board (IRB) Approval and Consent

It is expected that the IRB will have the proper representation and function in accordance with federally mandated regulations. The IRB will approve the consent form and protocol.

In obtaining and documenting informed consent, the investigator should comply with the applicable regulatory requirement(s) and should adhere to Good Clinical Practice (GCP) and to ethical principles that have their origin in the Declaration of Helsinki.

Before recruitment and enrollment onto this study, the subject will be given a full explanation of the study and will be given the opportunity to review and discuss the consent form and the Investigator or research staff (as appropriate) will answer any questions the subject has related to the study. Each consent form must include all the relevant elements currently required by the FDA Regulations and local or state regulations. Once this essential information has been provided to the subject and the investigator is assured that the subject understands the implications of participating in the study, the subject will be asked to give consent to participate in the study by signing an IRB approved consent form.

Prior to a subject's participation in the trial, the written informed consent form should be signed and personally dated by the subject and by the person who conducted the informed consent discussion.

9.3 Registration Procedures

All subjects must be registered by the UNC study coordinator before enrollment to study. Prior to registration, eligibility criteria must be confirmed with the UNC Study Coordinator.

9.4 Adherence to Protocol

Except for an emergency situation in which proper care for the protection, safety, and well-being of the study subject requires alternative treatment, the study shall be conducted exactly as described in the approved protocol.

9.4.1 Protocol Deviations/Violations

A protocol deviation is any unplanned variance from an IRB approved protocol that:

- Is generally noted or recognized after it occurs;
- Has no substantive effect on the risks to research participants;
- Has no substantive effect on the scientific integrity of the research plan or the value of the data collected;
- Did not result from willful or knowing misconduct on the part of the investigator(s).

An unplanned protocol variance is considered a violation if the variance meets any of the following criteria:

- Has harmed or increased the risk of harm to one or more research participants;
- Has damaged the scientific integrity of the data collected for the study;
- Results from willful or knowing misconduct on the part of the investigator(s);
- Demonstrates serious or continuing noncompliance with federal regulations, State laws, or University policies.

If a deviation or violation occurs, please follow the guidelines below:

Protocol Deviations: UNC will record the deviation in OnCore®, and report to any sponsor or data and safety monitoring committee in accordance with their policies.

Protocol Violations: Violations should be reported as per the IRB's requirements. They must also be recorded as a deviation in OnCore®.

Any events that meet the criteria for "Unanticipated Problems (UPs)" must also be reported to the IRB. Examples of such UPs include a lost or stolen laptop computer that contains sensitive study information. If the UP is the result of a protocol deviation, then the deviation must also be recorded in OnCore®.

9.5 Study Files and Record Retention

Study documentation includes all eCRFs, data correction forms or queries, source documents, Sponsor correspondence to Investigators, and regulatory documents (e.g., protocol and amendments, IRB correspondence and approval, signed subject consent forms).

Source documents include all recordings of observations or notations of clinical activities and all reports and records necessary for the evaluation and reconstruction of the clinical research study.

Government agency regulations and directives require that all study documentation pertaining to the conduct of a clinical trial must be retained by the study investigator. Study documents should be kept on file until three years after the completion and final study report of this investigational study.

9.6 Obligations of Investigators

The Principal Investigator is responsible for the conduct of the clinical trial at the site in accordance with Title 21 of the Code of Federal Regulations and/or the Declaration of Helsinki. The Principal Investigator is responsible for personally overseeing the treatment of all study subjects. The Principal Investigator must assure that all study site personnel, including sub-investigators and other study staff members, adhere to the study protocol and all FDA/GCP/NCI regulations and guidelines regarding clinical trials both during and after study completion.

The Principal Investigator at each institution or site will be responsible for assuring that all the required data will be collected and entered into the eCRFs. At the completion of the study, all eCRFs will be reviewed by the Principal Investigator and will require his/her final signature to verify the accuracy of the data.

9.7 Data and Safety Monitoring Plan

The Principal Investigator will provide continuous monitoring of subject safety in this trial with periodic reporting to the Data and Safety Monitoring Committee (DSMC).

Meetings/teleconferences will be held at a frequency dependent on study accrual. These meetings will include the investigators as well as study coordinators, data coordinators, regulatory associates, and any other relevant personnel the principal investigators may deem appropriate. At these meetings, the research team will discuss all issues relevant to study progress, including enrollment, safety, regulatory, data collection, etc.

The team will produce summaries or minutes of these meetings. These summaries will be available for inspection when requested by any of the regulatory bodies charged with the safety of human subjects and the integrity of data including, but not limited to, the oversight of Office of Human Research Ethics (OHRE) Biomedical IRB, the Oncology Protocol

Review Committee (PRC) or the North Carolina TraCS Institute Data and Safety Monitoring Board (DSMB).

The UNC LCCC Data and Safety Monitoring Committee (DSMC) will review the study on an annual basis at the time of IRB annual review, with the option to exempt from the study from future review. The UNC PI will be responsible for submitting the following information for review: 1) safety and accrual data; 2) significant developments reported in the literature that may affect the safety of subjects or the ethics of the study; 3) preliminary response data; and 4) summaries of team meetings that have occurred since the last report. Findings of the DSMC review will be disseminated by memo to the UNC PI, PRC, the UNC IRB and DSMB.

10 AUDITING AND MONITORING

The UNC LCCC will serve as the coordinating center for this trial. Data will be collected through a web based clinical research platform, REDCap. All data will be collected and entered into REDCap by research coordinators from UNC LCCC.

The site will provide direct access to source data/documents for trial-related monitoring, audits, IRB/IEC review, and regulatory inspection. As an investigator-initiated study, this trial may be audited. Any corrective and preventive action plans (CAPAs) resulting from the audit will be reviewed by the LCCC compliance committee for acceptability. All data will be monitored, and source data will be verified on selected subjects. Database queries will be issued on an ongoing basis on all subjects. The site should respond to data queries within 14 days of receipt.

11 AMENDMENTS

Should amendments to the protocol be required, the amendments will be originated and documented by UNC LCCC. It should also be noted that when an amendment to the protocol substantially alters the study design or the potential risk to the subject, a revised consent form may be required.

The written amendment, and if required the amended consent form, must be sent to UNC's IRB for approval prior to implementation.

12 STUDY DISCONTINUATION

Both Lineberger Comprehensive Cancer Center and the Principal Investigator reserve the right to terminate the study at the investigator's site at any time. Should this be necessary, Lineberger Comprehensive Cancer Center or a specified designee will inform the appropriate regulatory authorities of the termination of the study and the reasons for its termination, and the Principal Investigator will inform the IRB/IEC of the same. In terminating the study, Lineberger Comprehensive Cancer Center and the Principal Investigator will assure that adequate consideration is given to the protection of the subjects' interests.

13 CONFIDENTIALITY

All information generated in this study is considered highly confidential and must not be disclosed to any person or entity not directly involved with the study unless prior written consent is gained from Lineberger Comprehensive Cancer Center. However, authorized regulatory officials, IRB/IEC personnel, Lineberger Comprehensive Cancer Center and its authorized representatives are allowed full access to the records.

Identification of subjects and eCRFs shall be by initials, screening and treatment numbers only. If required, the subject's full name may be made known to an authorized regulatory agency or other authorized official.

14 REFERENCES

1. Coleman-Jensen A, Rabbitt MP, Gregory CA, Singh A. *Household Food Security in the United States in 2021*. U.S. Department of Agriculture Economic Research Service. Washington, D. C. 2022.
2. Mozaffarian D. Measuring and addressing nutritional secutiry to achieve health and health equity *Health Affairs Health Policy Brief. March 30,* 2023. https://doi.org/10.1377/hpb2023216.92558
3. American Academy of Pediatrics Council on Community Pediatrics. Poverty and child health in the United States. *Pediatrics.* 2016;137:e20160339. https://doi.org/10.1542/peds.2016-0339
4. Zenk S, Tabak L, Perez-Stable E. Research opportunities to address nutrition insecurity and disparities. *Journal of the American Medical Association.* 2022;327(20):1953-1954. https://doi.org/ 10.001/jama.2022.7159
5. Kelly A. Defining "serious illness". *Journal of Palliative Care Medicine* 2014; 17(9):985. https://doi.org/ 10.1089/jpm.2014.0164
6. Bona K, Blonquist T, Neuberg D, Silverman L, Wolfe J. Impact of socioeconomic status on timing of relapse and overall survival for children treated on Dana Farber Cancer Institute ALL Consortium Protocols (2000-2010). *Pediatric Blood & Cancer.* 2016;63(6):1012-1018. https://doi.org/10.1002/pbc.25928
7. Wadwa A, et al. Poverty and relapse risk in children with Acute Lymphoblastic Leukemia: Children's Oncology Group Study AALL03N1 report. *Blood.* 2023; 142(3):221-229. https://doi.org/10.1182/blood.2023019631
8. Bona K, et al. Poverty and targeted immunotherapy: survival in Children's Oncology Group clinical trials for high-risk neuroblastoma. J*ournal of the National Cancer Institute.* 2021;113(3):282-291. https://doi.org/10.1093/jnci/djaa107.
9. Barr RD, Gomez-Alvarez D, Jaime-Perez JC, Ruiz-Augiuelles GJ. Importance of nutrition in the treatment of leukemia and children and adolescents. *Archives of Medical Research* 2016;47:585-592. https://doi.org.10.1016/j.arcmed.2016.013
10. Tripodi SI, et. al. The importance of nutrition in children with cancer.*Tumori Journal.* 2023;109:19-27. https://doi.org.10.101177/
11. Berkowitz S, Palakshappa D, Seligman HK, Hanmer J. Changes in food insecurity and changes in patient-reported outcomes: a nationally representative cohort study. *Journal of General Internal Medicine.* 2021;37(14):3638-3644. https://doi.org.10.1007/s11606-021-07293-4
12. Pedretti, et al. Role of nutrition in pediatric patients with cancer. *Nutrients.* 2023;15:710. https://doi.org/10.3390/nu15030710
13. Orgel E, et al.. Caloric and nutrient restriction to augment chemotherapy efficacy for acute lymphoblastic leukemia: the IDEAL trial. *Blood Advances.* 2021;5(7):1853-1870. https://doi.org/10.1182/bloodadvances.20200004018
14. Bhatia S, et al. 6-MP adherence in a multiracial cohort of children with acute lymphoblastic leukemia: a Children's Oncology Group study. *Blood.* 2014;124(15):2345-2363. https://doi.org/10.1182/blood-2014-01-
15. Gomez-Laexander D, Ruiz-Augiuelles GJ, Ponce-De-Leon S. Nutritional status and socio-economic conditions as prognostic factors in the outsome of therapy in childhood

acute lymphophoblastic leukemia *International Journal of Cancer.* 1998; Supplement 11:52-55. https://doi.org/10.1002/(sici)1097-0215(1998)78:11+<52::aid-ijc15>3.0.co;2-3
16. Wilder ME, et al. The impact of social determinants of health on medication adherence: a systematic review and meta-analysis *Journal of General Internal Medicine.* 2021;36(5):1359-1370. https://doi.org/10.1007/s11606-020-06447-0
17. Gehle SC, et al. Accelerated epigentic aging and myopenia in yound adult cancer survivors. *Cancer Medicine.* 2023:12(11): 12149-12160. https://doi.org/10.1002/cam45908
18. Smitherman AB, et al. Accelerated aging among childhood, adolescent, and young adult cancer survivors is evidenced by increased expression of $p16^{INK4a}$ and frailty. *Cancer.* 2020;126:4975-4983.https://doi.org/10.1002/cncr.33112
19. Ogland-Hand C, Ciesielski TH, Daunov K, Beab MK, Nock NL. Food insecurity and nutritional challenges in adolescent and young adult cancer survivors in the USA: a narrative reveiw and call to action. *Nutrients.* 2023;15:1731. https://doi.org/10.3390/nu15071731
20. McDougall JA, Jaffe SA, Guest DD, Sussman AL. The balance between food and medical care: experiences of food security among cancer survivors and informal caregivers. *Journal of Hunger & Environmental Nutrition.* 2022;17(3):380-396. https://doi.org/10.1080/19320248.2021.1892295
21. Thom B, et al. Economic distress, financial toxicity, and medical cost-coping in young adult cancer survivors during the COVID-18 Pandemic: findings from an online survey. *Cancer.* 2021;127:4481-4491. https://doi.org/10.1002/cncr.33823
22. Nucci D, et al. Altered food behavior and cancer: a systematic review of the literature. *International Journal of Environmental Research & Public Health.* 2022;19:10299. https://doi.org/10.3390/iherph191610299
23. Umaretyia P, et al. PediaCARE: Development of a poverty-targeted intervention for pediatric cancer. *Pediatric Blood & Cancer.* 2021;68:e29195. https://doi/org/10.1002/pbc.29195
24. Downer S, Clippinger E, Kummer C. *Food is Medicine research action plan*. Published Jan. 27, 2022.
25. Hager K, et al. Association of national expansion of insurance coverage of medically tailored meaks with estimated hospitalizations and health care expenditures in the US. *JAMA Network Open* 2022;5(10):e2236898. https://doi.org/10.1001/jamanetworkopen.2022.36898
26. Nichol G, Hunt H. *The persistent and pervasive challenge of child poverty and hunger in North Carolina.* 2021; NC Poverty Research Fund UNC School of Law.
27. Bona K, et al. Prevalence and impact of financial hardship among New England pediatric stem cell transplantation families. *Biology of Bone Marrow Transplantation.* 2015;21(2):312-318. https://doi.org/10.1016/j.bbmt.2014.10.016
28. de Souza J, et al.. The development of a financial toxicity patient-reported outcome in cancer. *Cancer.* 2014;120:3245-3253. https://doi.org/10.1002/cncr.28814
29. Lansky S, et al. Childhood cancer: non-medical costs of the illness. *Cancer.* 1979;43:403-408. https://doi.org/10.1002/1097-0142(197901)43:1<403::aid-cncr2820430157>3.0.co;2-l
30. Santacroce S, Tan K, Killela M. Costs of illness to parents of children diagnosed with cancer. *European Journal of Oncology Nursing.* 2018; 35: 22-32.

31. Santacroce S, Kneipp S. A conceptual model of financial toxicity in pediatric oncology. *Journal of Pediatric Oncology Nursing*. 2018; 36(1): 6-16.
32. Berkowitz S, et al. Medically tailored meals for food insecurity and type 2 diabetes: Protocol for the Food as Medicine for Diabetes trial. *Contemporary Clinical Trials*. 2023; 124: 107039.
33. Berkowitz S, Fabreau G. Food insecurity: what is the clinician's role? *Canadian Medical Association Journal*. 2016;187(14):1031-1032.
34. Rodems R. *Hidden hardship in the United States. Material well-being above the poverty line*. 2019; Inequality Lab, University of Michigan
35. Gany F, et al. Food insecurity among cancer patients enrolled in the Supplemental Nutrition Program (SNAP). *Nutrition and Cancer.* 2021;73(2):206-214. https://doi.org/10.1080/01635581.2020.1743867
36. Keegan Wells D, et al. The care of my child with cancer: an instrument to measure caregiving demand in parents of children with cancer. *Journal of Pediatric Nursing.* 2002;17(3):201-210. https://doi.org/10.1053/jpdn.2002.124114
37. Cohen J, Goddard E, Brierley M-E, Bramley L, Beck E. Poor diet quality in children with cancer during treatment. *Journal of Pediatric Oncology Nursing.* 2021;38(5):313-321. https://doi.org/10.1177/1043454221011050
38. Kocamaz E, Gumus E, Akbayram S, Yazici A. Taste alteration in children with acute lymphoblastic leukemia undergoing maintenance treatment. *Journal of Pediatric Hematology Oncology*. 2022; 44:e1053-e1056.
39. Kamkhoad D, Santacroce S, Phonyiam R, Wang M. Symptom clusters that included gastrointestinal symptoms: a scoping review. *Oncology Nursing Forum*. 2023; 50(3):381-395.
40. van den Brink M, ter Hedde, van den Heuvel E, Tissing W, Havermans RC. The impact of changes in taste, smell and eating behaviors in children with cancer undergoing chemotherapy: a qualitative study. *Frontiers in Nutrition.* 2022; 9:984101.
41. Ijpma I, Renken RJ, ter Horst GJ, Reyners AKL. Metallic tast in cancer patients treated with chemotherapy. *Cancer Treatment Reviews*. 2015; 41: 179-186.
42. Webber T, Briata IM, DeCensi A, Cevassco I, Paleari L. Taste and smell disorders in cancer treatment: results from an integrative rapid systematic reveiw. *Molecular Sciences*. 2023; 24: 2538.
43. Santacroce S, Killela M. Khamkoad D, Leckey J, Hubbard G. He knew more than we wanted him to know: parent perceptions about their children's sense of pediatric cancer-related financial problems. *Pediatric Blood & Cancer*. 2021; 68(8): e29080.
44. Hager K, Kummar C, Lwin-Zwerdling A, Li Z. *Food is medicine research action plan. 2024*; Food & Society at the Aspen Institute.
45. Connolly C, Golden J. *Lessons ignored: children and pandemic. American Journal of Public Health*. 2023; 113(9):985-990.
46. Eldridge S, Chan C, et al. on behalf of the PAFS concensus group. CONSORT 2010 statement: extension to randomised pilot and feasibility trials. *BioMed Central*. 2016; 2(64): 1-32.
47. Nielsen J, Laundauer T. A mathematical model of usuability problems. Paper presented at: Proceedings of ACM INTERCHI'93 Conference. Amsterdam, The Netherlands.
48. Nielsen J. Estimating the number of subjects needed for a thinking aloud test. *International journal of Human Computer Studies*.1994; 41: 385-397.

49. Sick J, Hojer R, Olsen A. Children's self-reported reasons for accepting and rejecting food. *Nutrients.* 2019;11:2455. https://doi.org/10.3390/nu11102455
50. Dupuis L, et al. Validation of the Symptom Screeing in Pediatrics Tool in children receiving cancer treatments. *Journal of the National Cancer Institute.* 2018;110(6):661-668.
51. Kano T, Kanda K. Development and validation of a chemotherapy-induced taste alteration scale. *Oncology Nursing Forum*. 2013; 40(2):E79-85.
52. Weiner B, et al. (2017). Psychometric assessment of the three newly developed implementation outcome measures. *Implementation Science*, 12. Doi:10.1186/s13012-017-0635-3
53. U.S. Department of Agriculture Economic Research Service. U.S. *Household food security survey module: three-stage design, with screeners. 2012*. https://www.ers.usda.gov/topics/food-nutrition-assistabce/food-security-in-the-u-s-/measurement
54. Calloway E, Carpenter L, Gargano T, Sharp J, Yaorch A. Development of new measures to assess nutrition security and choice in dietary characteristics. *Appetite* 2022;179. https://doi.org/10.1016/j.appet.2022.206288
55. Hahn E, et al. PROMIS Cooperative Group (2014). New English and Spanish social health measures will facilitate evaluating health determinants. *Health Psychology,* 33(5), 490–499.
56. Beauchemin M, et al. Financial distress during treatment for pediatric acute lymphoblastic leukemia in the United States: A study protocol. *BMC Health Services. 2022; 22:832.* https://doi.org/10.1186/s12913-022-08201-0.
57. Prawitz A, et al.. InCharge Financial Distress/Financial Well-Being Scale: development, administration, and score interpretation. *Financial Counseling & Planning*. 2006;**17** 34-50 doi: doi:10.1037/t60365-000.
58. Bell ML, Whithead AL, Julious SA. Guidance for using pilot studies to inform the design of intervention trials with continous outcomes. *Clinical Epidemiolgy*. 2018;**10**: p. 153-157.
59. Brown R. On use of a pilot sample for sample size determination. *Statistics in Medicine,* 1995. **14**(17): p. 1933-1940.
60. Nevadal N, et al. Rapid versus traditional qualitative analysis using the Consolidated Framework for Implementation Research. *Implementation Science.* 2021;16:67.
61. Ponticorvo S, et al. Sex differences in the taste-evoked functional network. *Chemical Senses.* 2022 47:1-12.

15 APPENDICES

15.1 APPENDIX I – Names of Study Personnel

Sponsor:	Lineberger Comprehensive Cancer Center
Principal Investigator:	Sheila Judge Santacroce, PhD, RN, CPNP, FAAN<br>Beerstecher-Blackwell Distinguished Professor<br>School of Nursing<br>The University of North Carolina at Chapel Hill<br>South Columbia at Medical Drive<br>Email: sheila.santacroce@unc.edu<br>Phone: (919) 966-4298
Clinical Trials Office:	Lineberger Comprehensive Cancer Center<br>The University of North Carolina at Chapel Hill<br>450 West Drive, 3rd Floor,<br>Chapel Hill, NC 27599-7295<br>Phone: (919) 966-4432
Biostatistician:	Laura Farnan, MS, PhD<br>Lineberger Comprehensive Cancer Center -UCRF<br>Bioinformatics Building, 3162-K<br>Email: laura_farnan@med.unc.edu<br>Phone: (919) 482-4014

15.2 APPENDIX II – Study Measures

15.2.1 ADOLESCENT SURVEYS

15.2.1.1 Adolescent Medical Record Abstraction Form T1 (To be completed by study team)

1. Study ID#: __ __ __
2. Today's date: _ _/_ _/_ _
3. Diagnosis:
   a. Acute Lymphoid Leukemia
   b. Acute Myeloid Leukemia
   c. Hodgkin lymphoma
   d. Non-Hodgkin lymphoma
   e. Brain or other central nervous system cancer
   f. Neuroblastoma or other peripheral nerve tumor
   g. Hepatic tumor
   h. Osteosarcoma
   i. Ewing sarcoma
   j. Rhabdomyosarcoma
   k. Non-Rhabodoid sarcoma
   l. Germ cell or gonadal tumor
   m. Wilm's tumor
   n. Other (please specify): ___________________________
4. Diagnosis date: _ _ /_ _ /_ _
5. Treatment protocol number: _________________
6. Start date:
7. Chemotherapies received to date: (list) ____
8. Current cycle: _ _
9. Current cycle start date: _ _/_ _/_ _
10. Radiation therapy: No __ Yes__
11. If yes, radiotherapy field _____________________________________
12. Current height: _ _ _. _ centimeters
13. Current weight: _ _ _. _ kilograms

15.2.1.2 Adolescent Acceptability and Appropriateness

These questions ask your opinions about the Medically Tailored Meals for Kids (MTM-Kids) program. Please select the response that best fits your thoughts.

**Acceptable**	Completely disagree	Disagree	Neither agree nor disagree	Agree	Completely agree
The meal choices appeal to my family.					
We liked the variety of meal choices					
With MTM-Kids, our family felt my child's healthcare team cared about my family					
We recommend MTM-Kids					
**Appropriate**	Completely disagree	Disagree	Neither agree nor disagree	Agree	Completely agree
MTM-Kids is fitting for families like mine.					
10 meals per week is appropriate					
Weekly is the right timing for deliveries					
The meal size was suitable					

15.2.1.3 Adolescent Baseline

We want to ask some basic questions about you:

1. Your current age in years:

2. The sex assigned to you at birth
   a. Female
   b. Male
   c. Intersex
   d. Prefer not to answer

3. Your race (please select one):

a. American Indian or Alaskan Native
b. Asian
c. Black or African American
d. Hispanic or Latino
e. Middle Eastern or North African
f. Native Hawaiian or Other Pacific Islander
g. White
h. Prefer not to answer

4. The language you mainly use your home:
   a. English
   b. Spanish
   c. Other: ______________________

5. Your current grade (year) in school:
   a. 5th
   b. 6th
   c. 7th
   d. 8th
   e. 9th
   f. 10th
   g. 11th
   h. 12th
   i. Other: ____________________

15.2.1.4 Adolescent Alterations in Taste and Other Treatment-Related Symptoms

Thank you for agreeing to be in this research study. Our goal is to find ways to better support children and their families during the child's treatment with chemotherapy.

The first questions ask about taste changes some adolescents have after chemotherapy. Please click the circle that best describes how much that taste change **bothered you this week.**

	Not at all	A little	Medium	A lot	Extremely
Difficulty tasting sweet					
Difficulty tasting salty					
Difficulty tasting sour					
Difficulty tasting bitter					
Difficulty tasting flavors					
Everything tastes bad					
Food doesn't taste like it should					
Bitter taste in my mouth					
Bad taste in my mouth					
Metal taste in my mouth					
Can't eat hot food					

Can’t eat spicy food					
Can’t eat meat					

Please tell us about any other taste changes that bothered you this week by writing them here:
[insert text box]

The next questions are about other treatment-related symptoms adolescents can have. Please tell us how much each of these things **bothered you yesterday or today** by clicking the circle that best describes that amount:

	Not at all	A little	Medium	A lot	Extremely
Feeling disappointed or sad					
Feeling scared or worried					
Feeling cranky or angry					
Problems with thinking or remembering					
Changes in how your body or face look					
Feeling tired					
Mouth sores					
Headache					
Hurt or pain (other than headache)					
Tingly or numb hands or feet					
Throwing up or feeling like you might					
Changes in taste					
Constipation (hard to poop)					
Diarrhea (watery, runny poop)					

Please tell us about any other things that bothered you this week by writing them here:

15.2.2 PARENTAL SURVEYS

15.2.2.1 Food Insecurity

These statements are what people have said about their food situations. Please tell us whether the statement was often true, sometimes true, or never true for you in the last 6 months.

1. “The food that I bought just didn’t last, and I didn’t have money to get more.”
   a. Often true
   b. Sometimes true
   c. Never true
   d. Don’t know or prefer not to answer

2. “We couldn’t afford to eat balanced meals.”
   a. Often true
   b. Sometimes true
   c. Never true

d. Don't know or prefer not to answer

3. In the last 3 months, did you or other adults in your household ever cut the size of your meals or skip meals because there wasn't enough money for food?
   a. Yes
   b. No (Skip AD1a)
   c. Don't know or prefer not to answer (Skip AD1a)

[If YES above, then]
4. How often did this happen – almost every month, some months, but not every month, or in only 1 or 2 months?
   a. Almost every month
   b. Some months but not every month
   c. Only 1 or 2 months
   d. Don't know or prefer not to answer

5. In the last 3 months, did you ever eat less than you felt you should because there wasn't enough money for food?
   a. Yes
   b. No
   c. Don't know or prefer not to answer

6. In the last 3 months, were you ever hungry but didn't eat because there wasn't enough money for food?
   a. Yes
   b. No
   c. Don't know or prefer not to answer

15.2.2.2 Nutrition Insecurity

The following questions ask about your household's ability to decide what you eat
7. In the last 3 months, we had to eat foods that were not good for our health and well-being because we couldn't get other types of food
   a. Never
   b. Rarely
   c. Sometimes
   d. Often
   e. Always
   f. Don't know or prefer not to answer

8. In the last 3 months, we know there were things we should or should not eat for our health and wellbeing, but we could not get healthful food.
   a. Never
   b. Rarely
   c. Sometimes
   d. Often

   e. Always
   f. Don't know or prefer not to answer

9. In the last 3 months, we worried that the food we were able to eat would hurt our health.
   a. Never
   b. Rarely
   c. Sometimes
   d. Often
   e. Always
   f. Don't know or prefer not to answer

10. In the last 3 months, we had to eat the same thing for several days because we didn't have money to buy other food.
   a. Never
   b. Rarely
   c. Sometimes
   d. Often
   e. Always
   f. Don't know or prefer not to answer

11. In the last 3 months, we could control if we were able to eat quality food.
   a. Never
   b. Rarely
   c. Sometimes
   d. Often
   e. Always
   f. Don't know or prefer not to answer

12. In the last 3 months, we could control if were able to eat foods good for our health.
   a. Never
   b. Rarely
   c. Sometimes
   d. Often
   e. Always
   f. Don't know or prefer not to answer

15.2.2.3 Parent-Reported Acceptability, Appropriateness, Feasibility, Affordability, & Accessibility

These questions ask your opinions about the Medically Tailored Meals for Kids (MTM-Kids) program. Please select the response that best fits your thoughts.

**Acceptable**	Completely disagree	Disagree	Neither agree nor disagree	Agree	Completely agree
The meal choices appeal to our family					
We liked the variety of meal choices					
With MTM-Kids, our family felt my child's healthcare team cared about our family					
We recommend MTM-Kids					
**Appropriate**	Completely disagree	Disagree	Neither agree nor disagree	Agree	Completely agree
MTM-Kids is fitting for families like us					
10 meals per week is appropriate					
Weekly is the right timing for deliveries					
The meal size was suitable					
**Feasible**	Completely disagree	Disagree	Neither agree nor disagree	Agree	Completely agree
Ordering meals is easy					
Scheduling meal deliveries is doable					
The meals were simple to use					
The program helped me balance caring for my child with feeding my household					
**Affordable**	Completely disagree	Disagree	Neither agree nor disagree	Agree	Completely agree
The program eased our food expenses					
Having the meals readily available reduced our spending on take-out					
Having the meals on hand lessened our spending on eating out.					
**Accessible**	Completely disagree	Disagree	Neither agree nor disagree	Agree	Completely agree
MTM kids brought healthy meals to our door					
Healthy choices were readily available to my household with MTM-Kids					

15.2.2.4 Parent Contact Form

Please provide the following information for study-related communications

Your first name: Middle initial: Last name:

Mobile phone number (XXX) XXX -XXXX

Email address:

Preferred contact method for study reminders and surveys (check one):
____ Mobile phone ____ Email

Best days of the week to contact you (check all that apply)

___ Monday___ Tuesday ___ Wednesday ___ Thursday ___ Friday ___ Saturday ___ Sunday

Best time(s) of day to reach you (check all that apply)
___ Morning ___ Afternoon ___ Evening

Your home address
House number:
Street name:
City:
Zip code:

15.2.2.5 Financial Coping Strategies

Due to changes in your household expenses and work, have you done any of the following since your child was diagnosed (please select all that apply)?

	Never	Sometimes	Often	I don't have this
Use savings				
Use retirement funds				
Take out a loan or increase credit card debt				
Get another job, or increase work hours				
Not take a new job or opportunity because of worry about losing health insurance				
Receive money from fundraisers or crowdfunding				
Cut back on putting in retirement or education accounts				
Move to reduce mortgage or rent				
Cut back on spending for vacation, travel, or relaxation				
Cut back on spending for afterschool or summer activities for your children				
Cut back on spending for family celebrations				
Cut back on spending for groceries				
Cut back on spending for healthcare				
Cut back on spending for necessities for your family, such as clothing				
Cut back on spending for education				
Delay or skip filling prescriptions due to cost				
Skip or reduce medication doses due to cost				
Miss medical appointments due to cost				

Are other changes in how you manage your finances that you want to tell us about?

____

15.2.2.6 Financial Distress

The following questions are intended to get a sense of how financially secure you feel your family is. Please select the responses **most appropriate** for your situation.

41. What do you feel is the level of your financial stress today?

1	2	3	4	5	6	7
8	9	10				

Overwhelming stress   High stress   Low stress   No stress at all

42. On the stair steps below, mark (with an X) how satisfied you are with **your present financial situatio**n. "1" at the bottom represents complete dissatisfaction. "10" at the top represents complete satisfaction. The **more dissatisfied** you are, the lower the number you should mark. The **more satisfied** you are, the higher the number you should mark.

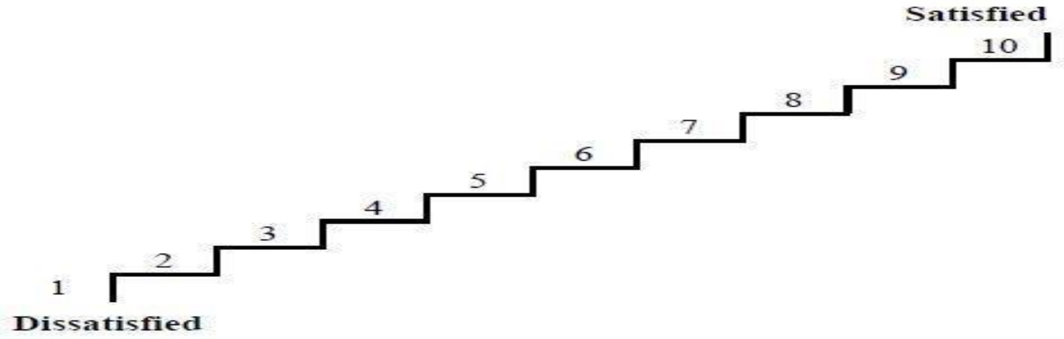

43. How do you **feel** about your **current financial situation**?

1	2	3	4	5	6	7	8	9	10

Feel overwhelmed   Sometimes feel worried   Not worried   Feel comfortable

44. How often do you worry about being **able to meet normal monthly living expenses**?

1	2	3	4	5	6	7	8	9	10

Worry All the time   Worry sometimes   Rarely worry   Never worry

45. How **confident** are you that you could find the money to pay for a **financial emergency** that costs about **$1,000?**

1	2	3	4	5	6	7	8	9	10

No Confidence   Little Confidence   Some Confidence   High Confidence

46. How **often** does this happen to you? You want to go out to eat, go to a movie or do something else and **don't go because you can't afford to**?

1	2	3	4	5	6	7	8	9	10

All the Time Sometimes Rarely Never

47. How **frequently** do you find yourself just getting by financially and living **paycheck to paycheck**?

1	2	3	4	5	6	7	8	9	10

All the Time Sometimes Rarely Never

48 How **stressed** do you feel about your personal finances **in general**?

1	2	3	4	5	6	7	8	9	10

All the Time Sometimes Rarely Never

15.2.2.7 Parent Socio-Demographics

Thank you for agreeing to be in this study. Our goal is to find ways to better support families during a child's treatment with chemotherapy. While we hope you will complete each item, if a specific question makes you feel uncomfortable, please select "prefer not to answer" when available, rather than leave it blank.

First, we will ask a few questions about you.

1. Your age in years _____

2. The sex assigned to you at birth (please select one)
   a. Female
   b. Male
   c. Intersex
   d. Prefer not to answer

3. Your race (please select one):
   a. American Indian or Alaska Native
   b. Asian
   c. Black or African American
   d. Hispanic or Latino
   e. Middle Eastern or North African
   f. Native Islander or Pacific Islander
   g. White
   h. Prefer not to answer

4. What language do you speak at home most of the time?
   a. English
   b. Spanish
   c. Other (please specify): ____________________

5. What language do you use for medical conversations about your child?
    a. English
    b. Spanish
    c. Other (please specify): ____________________

6. What is the highest level of schooling?
    a. Less than high school
    b. High school
    c. More than high school

7. Please select the option that best describes your current marital status:
    a. Married
    b. Living with a partner
    c. Single (living alone)
    d. Separated or divorced
    e. Widowed

8. What is your current employment status?
    a. Full-time (at least 30 hours per week)
    b. Part-time (less than 30 hours per week)
    c. Casual (as needed for short time periods)
    d. Not currently working for pay

9. To what extent have you or your child's other parent changed your employment situation because of child's diagnosis?
    a. one of us works more hours than before
    b. we both work same number of hours as before
    c. one of us is working fewer hours than before
    d. both of us are working fewer hours than before
    e. one of us quit work or took family leave

10. What type of insurance do you use to pay for your child's healthcare? (Select all that apply)
    a. Medicaid or state-funded low-income insurance program (e.g., CHIP)
    b. Private insurance (through work or school)
    c. Private insurance (purchased directly)
    d. Uninsured (self-pay)
    e. Other (please specify, such as Tri Care): ________________

11. How often do you need someone to help you read instructions or other written material from your or your child's health care team or social workers?
    a. Never
    b. Rarely
    c. Sometimes
    d. Often

e. Always

12. What is your relationship to the adolescent participating in this study with you?
    a. Parent
    b. Other legal guardian

The next questions are about your household.

13. Please provide your best estimate of your household's gross annual income (income before taxes are taken out). Include income from wages, child support, rental properties and social security, disability, veteran's benefits, unemployment benefits, worker's compensation and other money paid to you.
    a. $ ________________________
    b. I don't have a regular income
    c. Prefer not to answer

14. How many people does that income support?

______ Number of people

______ Not including the child receiving chemotherapy, how many of these people have health conditions that require extensive medical care, or help from you?

15.2.2.8 Study Eligibility Screener

We are developing a program to help us support better support families while their child is going through cancer therapy. May I ask you about your basic needs at home to see if you are eligible for this program?

Is your child between age 12 and 17.9 years?	YES	NO
Has your child received at least one cycle of chemotherapy?		
Are at least 2 more cycles of chemotherapy planned for your child?		
Has your child reported that food tastes different to them since they started chemotherapy?		
In the last 6 months, did you ever worry that the food would run out before you got money to buy more?		
If you checked YES, would you like help with this?		
In the last 6 months, has your utility company (gas, electric, oil, water) sent you a letter threatening to shut off service for not paying your bills?		
If you checked YES, would you like help with this?		
In the last 6 months was there a time when you couldn't pay the rent or mortgage in time because you didn't have the money?		
If you checked YES, would you like help with this?		

In the last 6 months, has lack of reliable transportation stopped anyone in your family from going to medical appointments, meetings, work, or getting things needed from daily living?		
If you checked YES, would you like help with this?		
Are any of your needs urgent? (for example: I don't have food for tonight, I don't have a place to sleep tonight).		

15.2.2.9 Time Demands

Over the past 7 days, how much time did you or someone else in your family spend cooking dinner or supper? Please do not include time spent eating. ______minutes total over 7 days

Please respond to each statement by marking the box that best describes you

	Not at all	Little bit	Some what	Quite a bit	Very much
I am satisfied with my ability to do things for my family					
I am satisfied with how much work I can do (including work at home)					
I feel good about my ability to do things for my family					
I am satisfied with my ability to do work that is important to me (including work at home)					
I am satisfied with my ability to participate in family activities					
I am satisfied with the amount of time I spend doing work (including work at home)					
I am happy with how much I do for my family					
I am satisfied with my ability to work (including work at home)					
The quality of my work is as good as I want it to be					
am satisfied with my ability to do caregiving tasks.					
I am satisfied with my ability to meet the needs of my family					
I am satisfied with my ability to perform my daily routines					

15.3 APPENDIX III – Study Semi-Structured Interview Guides

15.3.1 Parent Interview Guide

Thank you for taking the time to speak with me today about the MTM Kids research study, which is looking at a new way of supporting families' food-related needs during treatment. We need your feedback on what parts of MTM Kids have worked well and not so well for you over the last month. We also appreciate your advice about what you liked, did not like, and/or we should change about MTM Kids to make it better for future families. We value your input and willingness to be in this study.

I am part of the MTM Kids research team. I have no clinical involvement with the healthcare team taking care of your child. My goal for talking with you today is to find out what about MTM Kids is not working and what is working so the study team can improve MTM Kids. Nothing you share will have effects on the care your child is receiving or your ability to continue in this study.

To start, I want to get a better understanding of your experience with MTM Kids.

1. When you think about MTM Kids, what words would you use to describe it?

2. The goal of MTM Kids is to help families meet their needs for nutritious, easy to prepare food during a child's cancer treatment. From your perspective, how well is MTM Kids doing that? **Rephrase options:** Can you describe any ways in which MTM Kids has been useful (or not useful) to you and your family? **Probe on:**

   a. What changes would you like to see made to MTM Kids to make it better?
   b. [if not mentioned] What about the number of meals per week?

3. When you think about your family's needs right now for healthy, easy to prepare food right now, can you describe how MTM Kids does and doesn't help address those needs? **Probe on:**

   a. What specific changes would you like to see in MTM Kids to make it better?
   b. About how often you are placing an MTM Kids order?

[If less often than weekly] What influenced your ordering meals less often than weekly? **Probe on:**

a. Number of meals, meal size/portions, meal types, freezer space?
b. How difficult or easy has it been to order meals online?

[If number of meals, portions, types, space] what ideas do you have about how we could to improve X
[If difficult] please walk me through aspects of the ordering process that were difficult for you

4. How easy or difficult was it for you to select meals that would appeal to your family?

[If difficult], Please walk me through some aspects of meal selection that were difficult for you.
Probe on:

a. meal types

5. How easy or difficult was receiving meal deliveries for you?

[If difficult], Please walk me through some difficulties with meal deliveries.

6. How would you describe the overall influence that MTM Kids is having on your household's basic needs (food, transportation, utilities, housing).

7. You mentioned [insert barriers mentioned]; any other difficulties you using MTM Kids?
Probe on:

a. What other factors that prevented you from getting meals when you needed them?
b. What other factors that prevented you from using MTM Kids how you wanted?

8. Would you participate in MTM Kids again or recommend it to another family? What would you say to that family?

The next questions ask about the connections you see, if any, between MTM Kids and the overall care that your child is receiving.

9. From your perspective, how valuable or not is MTM Kids in connection to clinical care for your child?

10. When in their child's illness do you think MTM Kids be most helpful to families? **Probe**: around diagnosis, after hospitalization, transplant, or another type of major illness-related event

11. What else do you want to say about your experience with MTM Kids and needed improvements?

Again, thank you for taking the time to speak with me today about the MTM Kids research study. We value your input and willingness to be in this study.

15.3.2 Adolescent Interview Guide

Thank you for talking with me today about the MTM Kids research study – that's the frozen meals being delivered to your family. We want to find ways to help kids going through chemo and their families have meals that are easy to prepare. You are in the MTM-Kids study, which is looking at a new way of helping families during treatment. We want to make the MTM program works for kids like you. We ask for your thoughts about one of the frozen meals you tried. We also ask for your advice about how to make the frozen meals better for kids and their families. We value your input and saying yes to this study.

I am part of the MTM Kids research team. I'm not a part of the team taking care of you. I am talking with you to learn what about MTM Kids is and is not working for kids going through chemo so the study team can make the meals better for kids.

1. To start, please show me or take a share a picture/show us a MTM-Kids meal you tried. **Probe on**:

[if they haven't tried any] Please tell me about why you haven't tried a meal yet. Now, please tell me about something else you ate recently.

2. How did that meal/food work for you? **Probe on**:

a. How was the taste for you – too spicy, not spicy enough?
b. How was the smell?
c. How was the amount – too much, not enough?
d. Did someone heat it up for you, or did you do it yourself?

[if by self] How was that for you?

3. What are your ideas about how we can make the frozen meals better for kids going through chemo? **Probe on**:

a. More /less spice?
b. Smaller/larger portions?
c. Add more meal types?

[if more types] What ideas do you have about new meals or other things to eat that kids going through chemo would enjoy?

4. When you think about your family's needs right now for healthy, easy to prepare food right now, can you describe how MTM Kids does and doesn't help address those needs? **Probe on:**

c. What specific changes would you like to see in MTM Kids to make it better?
d. Who is ordering the MTM Kids meals?

[if the adolescent]

a. About how often are you doing that?

[If less often than weekly] What influenced your ordering meals less often than weekly? **Probe on:**

c. Number of meals, meal size/portions, meal types, freezer space?
d. How difficult or easy has it been to order meals online?

[If number of meals, portions, types, space] what ideas do you have about how we could to improve X
[If difficult] please walk me through aspects of the ordering process that were difficult for you

5. How easy or difficult was it for you to select meals that would appeal to you and your family?

[If difficult], Please walk me through some aspects of meal selection that were difficult for you. **Probe on**:

b. meal types

6. How easy or difficult was receiving meal deliveries for you?

[If difficult], Please walk me through some difficulties with meal deliveries.

7. Would you participate in MTM Kids again or recommend it to another family? What would you say to that family?

The next questions ask about the connections you see, if any, between MTM Kids and the overall care that you are receiving.

8. From your perspective, how valuable or not is MTM Kids in connection to your healthcare?

9. When do you think MTM Kids be most helpful to families like yours? **Probe**: around diagnosis, after hospitalization, transplant, or another type of major illness-related event

10. What else do you want to tell me about your experience with the MTM Kids study and how we can make the frozen meal offerings better for kids like you?

Again, thank you for talking with me today about the MTM Kids research study. We value your ideas and saying yes to this study.
