# Supplementary material for: Study protocol: Feasibility of medically tailored meals for pediatric populations at risk for disparities in serious illness outcomes due to inequities in food-related social drivers of health (MTM-Kids)
Source: PLoS One. 2025 Jul 31;20(7):e0326762. doi: 10.1371/journal.pone.0326762 (PMC12312939; doi:10.1371/journal.pone.0326762)
Supplement: S3 File — (DOC) [file pone.0326762.s003.doc]

|  | **Enrollment** | **Study Period** | | | | **Close-out** |
| --- | --- | --- | --- | --- | --- | --- |
| **TIMEPOINT** | ***-t_1_*** | ***t_1_*** | ***t_2_*** | ***t_3_*** | ***t_4_*** | ***t_x_*** |
| **ENROLLMENT:** |  |  |  |  |  |  |
| **Eligibility screen** | X |  |  |  |  |  |
| **Informed consent** | X |  |  |  |  |  |
| **INTERVENTION:** |  |  |  |  |  |  |
| ***MTM-Kids*** |  |  |  |  |  |  |
| **ASSESSMENTS^1^:** |  |  |  |  |  |  |
| ***SDOH not targeted by intervention*** |  | X | X | X | X |  |
| ***SDOH targeted by intervention*** |  | X |  |  | X |  |
| ***Nutritional Pathway*** |  | X | X | X | X |  |
| ***Cost-coping Pathway*** |  | X |  |  | X |  |
| ***Time Demands Pathway*** |  | X |  |  | X |  |
| ***Household Financial Well-Being*** |  | X |  |  | X |  |
| ***Intervention Feasibility*** |  |  | X | X | X |  |
| ***Feasibility of Future Efficacy Trial*** |  |  |  |  |  | X |

1 See Table 1 for specific measures within each domain
